# Supplementary material for: Differential and shared genetic effects on kidney function between diabetic and non-diabetic individuals
Source: Commun Biol. 2022 Jun 13;5:580. doi: 10.1038/s42003-022-03448-z (PMC9192715; doi:10.1038/s42003-022-03448-z)
Supplement: Supplementary file 2 — Supplementary Information [file 42003_2022_3448_MOESM2_ESM.pdf]

Supplementary Material – Winkler et al.

Contents

Supplementary Material – Winkler et al. 1

Supplementary Table 1. Sensitivity analyses without log transformation on eGFR for 7 difference locus lead variants.. 3

Supplementary Table 2. GCTA-analysis yields 2 loci with significant secondary joint test signals. 4

Supplementary Table 3. Human kidney phenotype for genes at 32 novel eGFR loci. 5

Supplementary Figure 1. Three eGFR loci were identified with differential effects by diabetes status in stage 1 and replicated in stage 2 6

Supplementary Figure 2. Unconditioned and conditioned difference P Values for the *UMOD/PDILT* locus. 7

Supplementary Figure 3. Distribution of difference P values in stage 1+2 combined.......... 8

Supplementary Figure 4. Unconditioned and conditioned regional difference P-values for the seven identified difference loci in stage 1+2. 9

Supplementary Figure 5. Stage 1 and stage 2 effect sizes for eGFR by DM and noDM for the 11 differential loci. 11

Supplementary Figure 6. Power to detect DM/noDM-differential effects for eGFR for varying effect sizes in DM and noDM. 12

Supplementary Figure 7. Distribution of joint, DM-only and noDM-only P-values for eGFR. 13

Supplementary Figure 8. Gene priotisation (GPS) at the seven identified difference loci. .14

Supplementary Figure 9. Gene prioritization at the 32 novel eGFR loci yields 22 genes. ..15

Supplementary Note 1. Details on stage 1 overall+difference test approach. 16

Supplementary Note 2. Robustness of the observed differences. 16

Supplementary Note 3. Power considerations....17

Supplementary Note 4. Extended study acknowledgments....18

Supplementary Note 5. VA Million Veteran Program: Core Acknowledgement for Publications (Updated April 6, 2020)....36

Supplementary Note 6. LifeLines group author genetics....38

References....39

Supplementary Table 1. Sensitivity analyses without log transformation on eGFR for 7 difference locus lead variants.
Shown are DM-status specific meta-analysis results based on eGFR (without log-transformation) including individuals from Uk Biobank and MVP. Results are shown for the seven difference variants.

						DM (UKB + MVP)				noDM (UKB + MVP)				
**locusid**	**rsid**	**Gene**	**ea**	**oa**	**eaf (UKB,DM)**	**beta**	**se**	**p**	**n**	**beta**	**se**	**p**	**n**	**Pdiff**
d1	rs77924615	*UMOD-PDILT*	G	A	0.80	-2.02	0.11	1.5E-74	77,047	-0.99	0.030	1.3E-241	539,930	**1.4E-19**
d2	rs434215	*TPPP*	A	G	0.27	-0.94	0.10	1.9E-19	77,047	-0.38	0.029	3.5E-41	539,930	**2.8E-07**
d3	rs55722796	*MED1-NEUROD2*	C	T	0.26	7.7E-03	0.17	0.96	19,617	0.52	0.029	4.9E-71	416,964	**3.3E-03**
d4	rs1828678	*CSRNP1*	C	G	0.67	0.35	0.092	1.6E-04	77,047	-0.056	0.025	0.025	539,930	**2.4E-05**
d5	rs963837	*DCDC5*	T	C	0.54	-0.78	0.089	1.8E-18	77,047	-0.47	0.024	2.6E-87	539,930	**6.6E-04**
d6	rs1882963	*NRIP1*	G	C	0.78	0.71	0.11	2.9E-11	77,047	0.24	0.028	2.7E-17	539,930	**2.1E-05**
d7	rs2619264	*SLC22A2*	G	A	0.24	-0.44	0.10	2.7E-05	77,047	-0.16	0.028	4.3E-09	539,930	**0.011**

Supplementary Table 2. GCTA-analysis yields 2 loci with significant secondary joint test signals.
Shown are independent index variants at joint test loci with secondary signals idntified by GCTA based on the EUR-only combined stage results. Abbreviations: bC: conditoned effect size; seC: conditioned standard error; pC: conditioned P value; ea: effect allele; pdiffC: conditioned difference P; pjointC: conditioned joint P

							DM				noDM					
**locusid**	**signalid**	**SNP**	**SNP_conditioned_on**	**Chr**	**Pos (b37)**	**ea**	**eaf**	**bC**	**seC**	**pC**	**eaf**	**bC**	**seC**	**pC**	**pdiffC**	**pjointC**
js1	1	rs34218958	rs7574806	2	64895903	T	0.81	0.0046	0.0013	4.3E-04	0.80	0.0023	3.3E-04	1.6E-12	0.085	2.9E-14
js1	2	rs7574806	rs7596561[a]	2	64894056	T	0.83	0.0138	0.0032	1.1E-05	0.83	0.0072	0.0017	3.4E-05	0.066	1.2E-08
js16	1	rs7383876	rs12705390	7	105938237	A	0.74	-0.0020	0.0013	0.12	0.74	-0.0020	3.3E-04	7.1E-10	0.98	1.7E-09
js16	2	rs12705390	rs7383876	7	106410777	A	0.21	-0.0054	0.0012	9.0E-06	0.21	-0.0013	3.3E-04	5.5E-05	**0.001**	1.5E-08

[a] r^2=0.98 to rs34218959

Supplementary Table 3. Human kidney phenotype for genes at 32 novel eGFR loci.
For the candiate genes mapping to the 32 novel eGFRcrea loci, the table shows human kidney-related diseases or phenotypes found in Online Mendelian Inheritance in Man (OMIM) or Groopman et al [1].

locus_id	Gene	Disease / Phenotype	Source	Chr	Start_of_gene	End_of_gene
js4	*ALPL*	HYPOPHOSPHATASIA, INFANTILE | HYPOPHOSPHATASIA, INFANTILE	OMIM; Groopman et al	1	21835857	21904905
js4	*CDC42*	TAKENOUCHI-KOSAKI SYNDROME; TKS	OMIM	1	22379119	22419436
js4	*HSPG2*	Schwartz-Jampel syndrome	Groopman et al	1	22148724	22263790
js5	*KANK4*	Nephrotic syndrome	Groopman et al	1	62701836	62785083
js9	*SLC2A2*	FANCONI-BICKEL SYNDROME; FBS | FANCONI-BICKEL SYNDROME; FBS	OMIM; Groopman et al	3	170714136	170744768
js11	*SLC30A9*	BIRK-LANDAU-PEREZ SYNDROME; BILAPES	OMIM	4	41992522	42089551
js13	*PPA2*	SUDDEN CARDIAC FAILURE, INFANTILE; SCFI	OMIM	4	106290233	106395227
js18	*EYA1*	OTOFACIOCERVICAL SYNDROME 1; OTFCS | BRANCHIOOTORENAL SYNDROME 1; BOR1	OMIM; Groopman et al	8	72109667	72274467
js19	*CUBN*	Megaloblastic anemia 1-finnish type	Groopman et al	10	16865964	17171816
js20	*RAB18*	Micro syndrome	Groopman et al	10	27793102	27831166
js22	*HPS1*	Hermansky-Pudlak syndrome 1	Groopman et al	10	100175954	100206704
js22	*HPSE2*	UROFACIAL SYNDROME 1; UFS1 | UROFACIAL SYNDROME 1; UFS1	OMIM; Groopman et al	10	100216833	100995632
js24	*C2CD3*	OROFACIODIGITAL SYNDROME XIV; OFD14	OMIM	11	73723758	73882064
js26	*BRCA2*	WILMS TUMOR 1; WT1 | FANCONI ANEMIA, COMPLEMENTATION GROUP D2; FANCD2	OMIM	13	32889616	32973809
js28	*SLC7A7*	LYSINURIC PROTEIN INTOLERANCE; LPI | LYSINURIC PROTEIN INTOLERANCE; LPI	OMIM; Groopman et al	14	23242431	23289020
js31	*FAT4*	HENNEKAM LYMPHANGIECTASIA-LYMPHEDEMA SYNDROME 2; HKLLS2 | VAN MALDERGEM SYNDROME 2; VMLDS2	OMIM; Groopman et al	4	126237566	126414087

Supplementary Figure 1. Three eGFR loci were identified with differential effects by diabetes status in stage 1 and replicated in stage 2

We searched for DM/noDM-differential genetic associations on eGFR using the difference test approach and the overall+difference approach in stage 1 (CKDGen and UKB, n_{DM}=109,993, n_{noDM}=1,070,999). Three difference loci were identified and replicated. a) Shown are difference test P-values over chromosomal base position (Manhattan plot) highlighting the one identified by the difference test approach (red, $P_{Diff}<5x10^{-8}$) and the two loci identified by the overall+difference test approach (orange, $P_{Diff}<8.2x10^{-5}$=0.05/610, corrected for 610 with stage 1 $P_{Overall}<5x10^{-8}$ as published previously [2]). Loci are annotated by the name(s) of the nearest gene(s). b) Shown is the distribution of observed versus expected DM/noDM-difference P-values (QQ plot). The genomic control inflation factor for the stage 1 difference test was λ_{GC}=1.04.

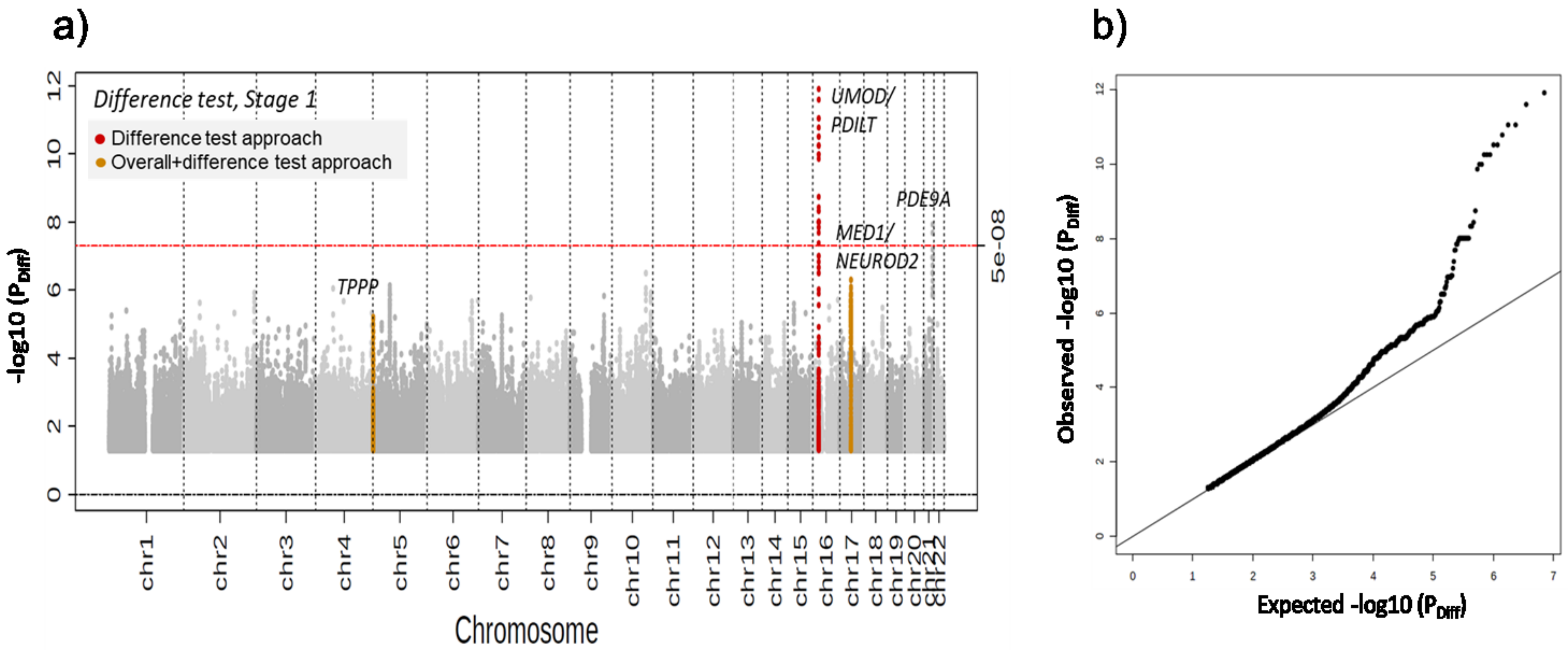

Supplementary Figure 2. Unconditioned and conditioned difference P Values for the *UMOD/PDILT* locus.

We computed difference P-Values at the *UMOD/PDILT* locus unconditioned and conditioned for the two previously identified independent index variants rs77924615 or rs34882080 [3], respectively, in stage 1 (n_{DM}=109,993, n_{noDM}=1,070,999). Shown are difference P-values in regional association plots for each variant in *UMOD-PDILT* locus. a) color-coding correlation to rs79924615 unconditioned (upper panel) and conditioned on rs34882080 (lower panel); and b) color-coding correlation to rs34882080 unconditioned (upper panel) and conditioned on rs79924615 (lower panel). This highlights that the rs77924615 showed a significant difference even after conditioning for rs34882080 but the rs34882080 difference disappeared after conditioning for rs77924615.

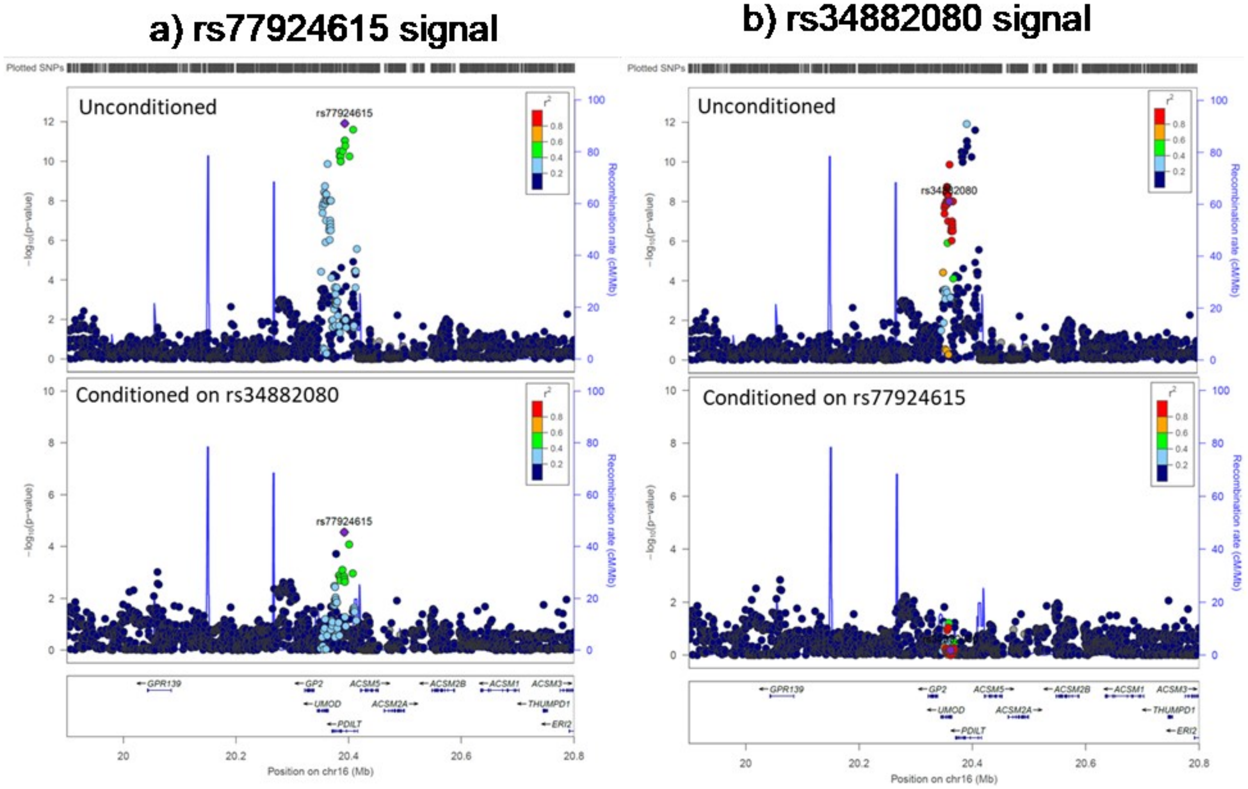

Supplementary Figure 3. Distribution of difference P values in stage 1+2 combined.

Shown is observed difference P-values versus expected (QQ plot) for the combined stages 1+2 (n_{DM}=178,691; n_{nodM}=1,296,113). The genomic control inflation factor for the stage 1+2 difference test was again λ_{GC}=1.04.

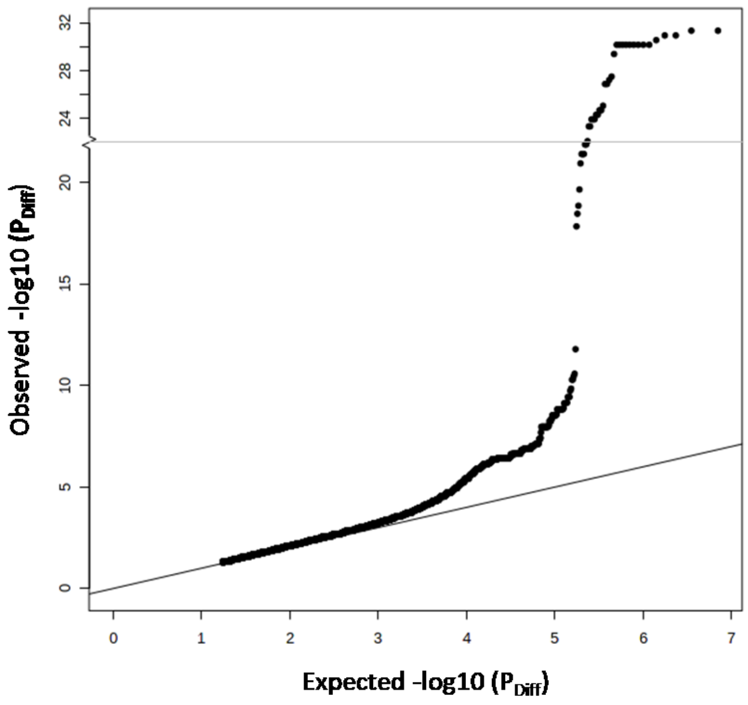

Supplementary Figure 4. Unconditioned and conditioned regional difference P-values for the seven identified difference loci in stage 1+2.

We computed difference P-values for each of the seven identified difference loci unconditioned and conditioned on the respective lead variant based on combined stage 1+2 (n_{DM}=178,691; n_{nodM}=1,296,113). Shown are unconditioned and conditioned difference P-values for the locus near a) *UMOD/PDILT*, conditioned on stage 1 lead variant rs77924615; b) *UMOD/PDILT*, conditioned on stage 1+2 lead variant rs9928003; c) *TPPP*; d) *MED1-NEUROD2*; e) *CSRNP1*; f) *DCDC5*; g) *NRIP1*, and h) *SLC22A2*.

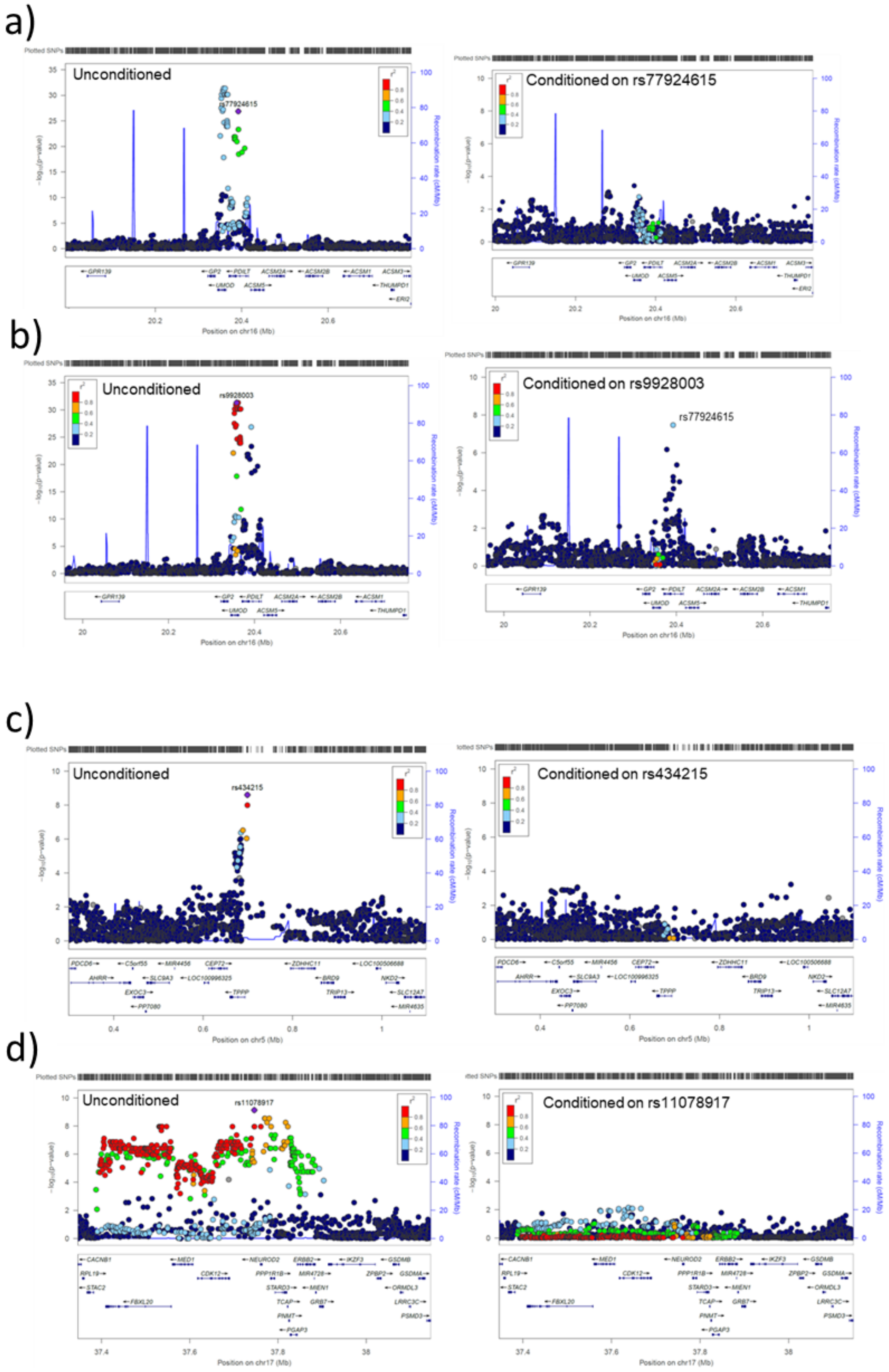

e)

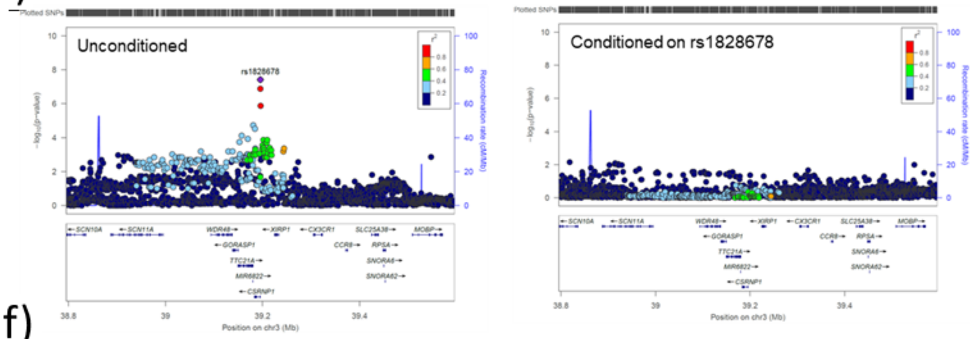
Unconditioned
rs1828678
Conditioned on rs1828678
Position on chr3 (Mb)

f)

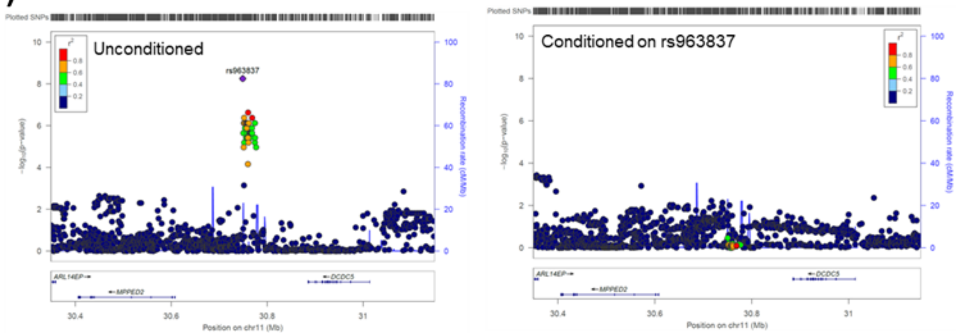
Unconditioned
rs963837
Conditioned on rs963837
Position on chr11 (Mb)

g)

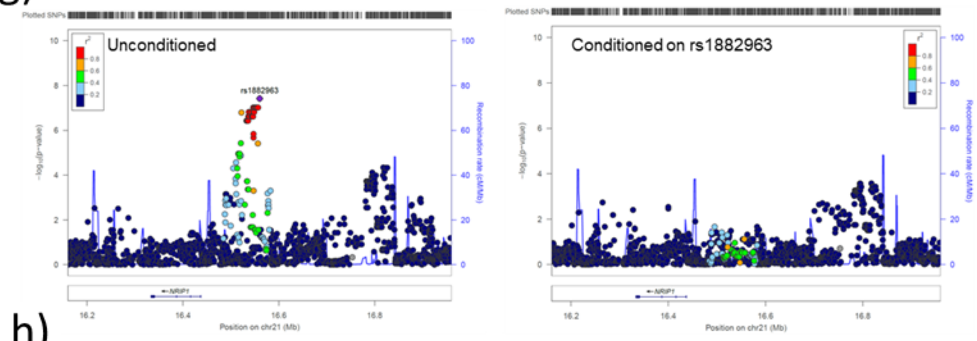
Unconditioned
rs1882963
Conditioned on rs1882963
Position on chr21 (Mb)

h)

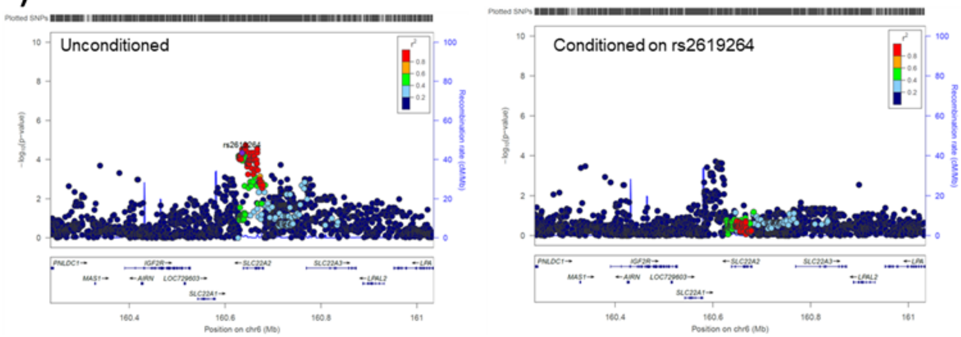
Unconditioned
Conditioned on rs2619264
Position on chr6 (Mb)

Supplementary Figure 5. Stage 1 and stage 2 effect sizes for eGFR by DM and noDM for the 11 differential loci. Shown are comparisons of effect sizes from stage 1 (n_{DM}=109,993; n_{nodM}=1,070,999) and stage 2 (n_{DM}=68,698, n_{NoDM}=225,114) for a) individuals with DM; or b) individuals with noDM. Indicated in red are the 7 loci identified by the difference screens (from **Table 1**, established difference) and in black the 4 additional novel eGFR loci with suggestive difference (from **Table 2**). Error bars reflect 95% confidence intervals of the estimated genetic effect.

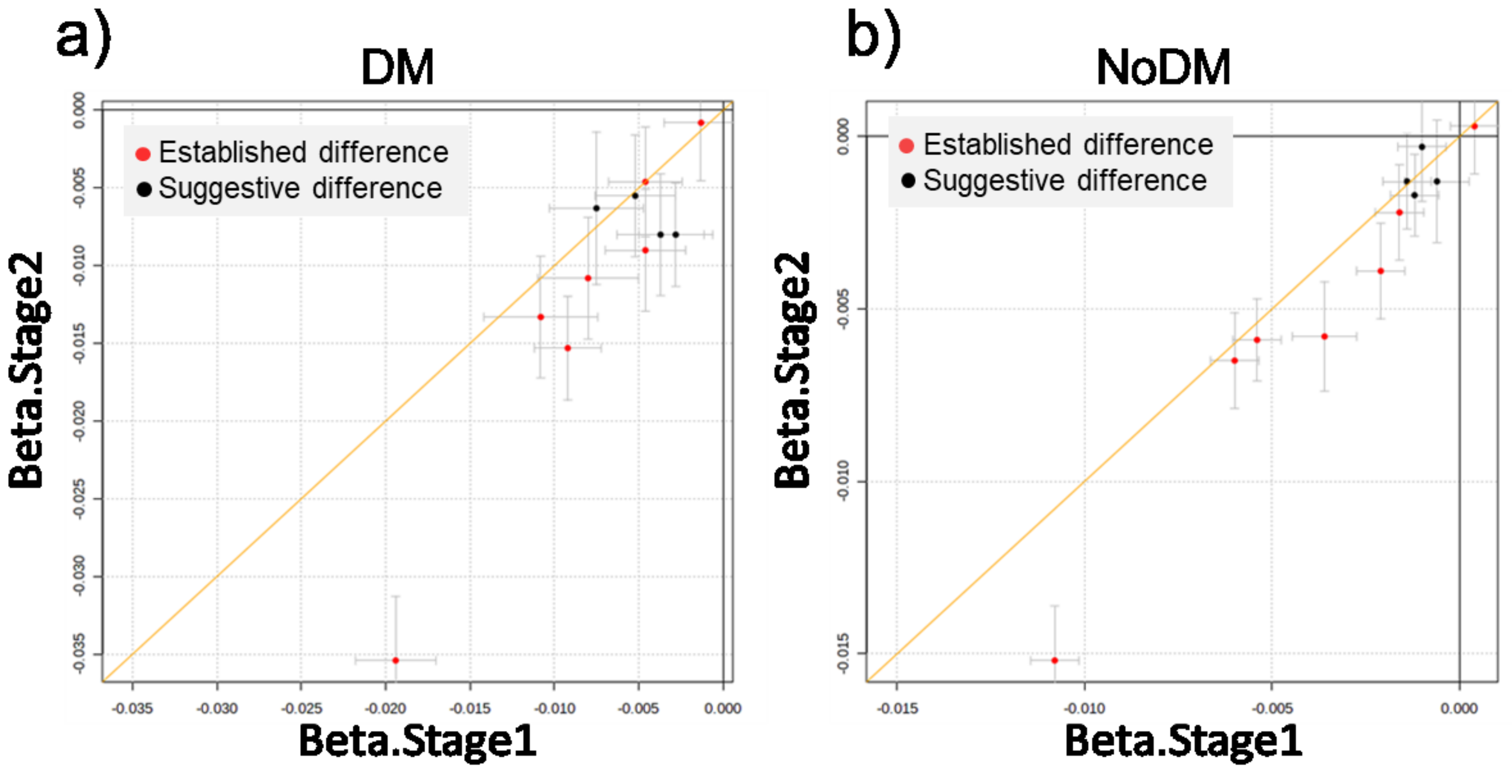

Supplementary Figure 6. Power to detect DM/noDM-differential effects for eGFR for varying effect sizes in DM and noDM.
Based on our stage 1+2 sample size of ~180,000 individuals with DM and ~1,300,000, we computed the power to identify DM/noDM-differential eGFR associations by the difference test approach or the overall+difference test approach for varying log eGFR effect sizes in DM (x-axis) and noDM (y axis). Shown are heatmaps indicating the power (color code) for the respective DM-/noDM effect size for three different minor allele frequencies (MAF). a) common variant (30% minor allele frequency, MAF), b) low frequency variant (5% MAF), and c) rare variant (1% MAF). d) Six scenarios of DM-only or noDM-only effect sizes are highlighted and respective power estimates are given; these scenarios are marked in the heatmaps by different symbols.

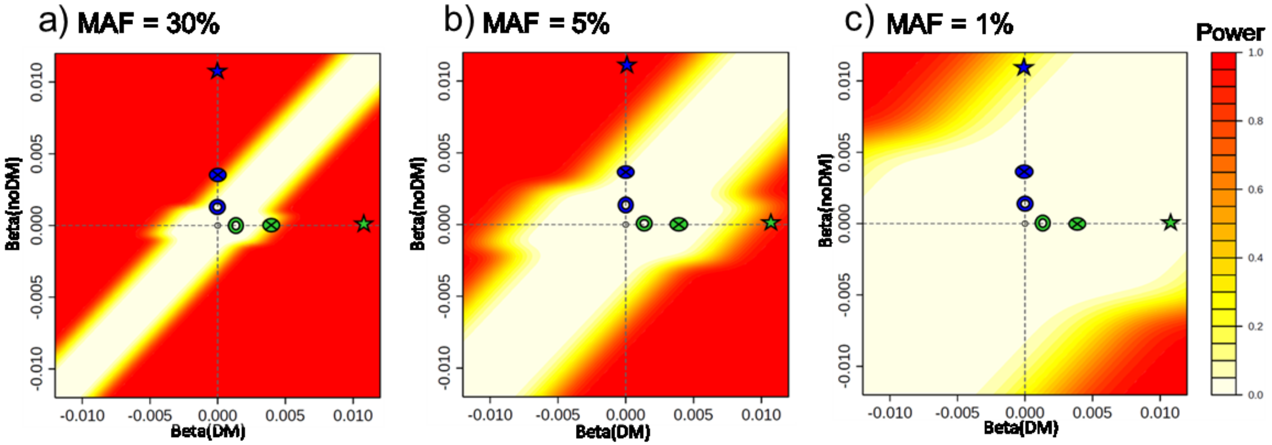

d)

	Beta		Power			
Symbol	DM	noDM	AF = 30%	AF = 5%	AF = 1%	Comment
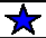	0	0.011	>99%	>99%	19%	Large effect in noDM (~*UMOD/PDILT* effect), zero effect in DM
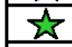	0.011	0	>99%	88%	<1%	Large effect in DM (~*UMOD/PDILT* effect), zero effect in noDM
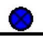	0	0.004	92%	6%	<1%	Medium effect in noDM, zero effect in DM
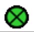	0.004	0	35%	<1%	<1%	Medium effect in DM, zero effect in noDM
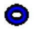	0	0.001	<1%	<1%	<1%	Small effect in noDM, zero effect in DM
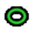	0.001	0	<1%	<1%	<1%	Small effect in DM, zero effect in noDM

Supplementary Figure 7. Distribution of joint, DM-only and noDM-only P-values for eGFR.
Shown are the distributions of observed versus expected P-values (QQ plots) from the joint test (black), noDM-only test (blue) and DM-only test (green) for a) stage 1 (n_{DM}=109,993, n_{noDM}=1,070,999) and b) combined stage 1+2 (n_{DM}=178,691; n_{nodM}=1,296,113).

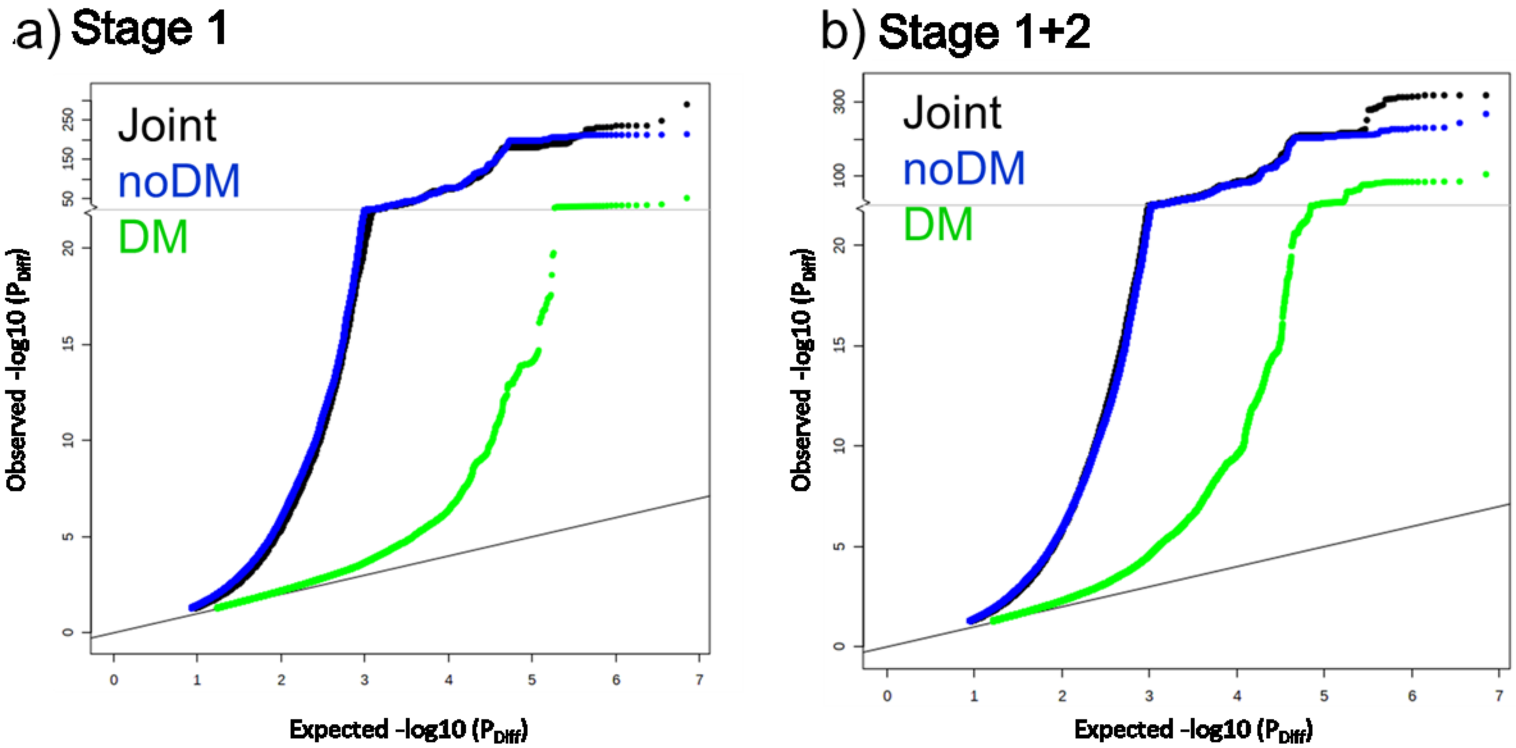

Supplementary Figure 8. Gene priotisation (GPS) at the seven identified difference loci.

By querying the GPS by Stanzick *et al* [2], we identified 7 genes located at 6 of the 7 difference loci that were mapping to a credible variant that was deleteriously protein-relevant within the gene (CADD≥15 [4]), to a credible variant that was an eQTL in kidney tissue [5,6], or genes that were known for kidney-related phenotypes in human (OMIM [7], or Groopman et al. [1]). For presentation in the main manuscript (**Figure 6**), the list of genes with evidenced kidney phenotype was manually reviewed and reduced.

Abbreviations: Position=b37

								99% credible variants within the gene with CADD ≥ 15			99% credible variants within the locus				Evidenced kidney phenotype
											eQTL			sQTL	
							Weights:	1	1	1	1	1	1	1	1
Locus name	Gene	distance to difference variant	Chr of gene	Position start of gene	Position end of gene	credible variants in the signal	Score	stop-gained/ stop-lost/ non-synonymus	canonical-splice/ noncoding-change/ synonymous/ splice-site	other	NEPTUNE glomerulus	NEPTUNE tubulointerstitium	GTEx kidney	GTEx kidney	Human
[UMOD/PDILT]	*UMOD*	-28132	16	20344372	20364200	1	1	0	0	0	0	0	0	0	1
[TPPP]	*SLC6A19*	502663	5	1201709	1225230	2	1	0	0	0	0	0	0	0	1
[TPPP]	*TPPP*	0	5	659976	693510	2	1	0	0	0	0	2	0	0	0
[MED1/NEUROD2]	*PGAP3*	271747	17	37827374	37844323	201	1	0	0	0	0	199	0	0	0
[MED1/NEUROD2]	*CDK12*	62136	17	37617763	37690818	201	1	0	0	1	0	0	0	0	0
[DCDC5]	*PAX6*	1057249	11	31806339	31839509	3	1	0	0	0	0	0	0	0	1
[NRIP1]	*NRIP1*	-122992	21	16333555	16437126	5	1	0	0	0	0	0	0	0	1
[SLC22A2]	*PLG*	487966	6	161123224	161175085	80	2	0	0	0	0	7	0	0	1
[SLC22A2]	*LPA*	317256	6	160952514	161087407	80	1	0	0	0	0	7	0	0	0
[SLC22A2]	*SLC22A1*	-55508	6	160542862	160579750	80	1	1	0	0	0	0	0	0	0
[SLC22A2]	*SLC22A2*	2535	6	160637793	160679963	80	1	0	1	0	0	0	0	0	0

Supplementary Figure 9. Gene prioritization at the 32 novel eGFR loci yields 22 genes. By querying the GPS derived for the novel eGFR loci according to the approach described previously [2] for the 371 genes at the 32 novel loci (**Supplementary Data 11**), we identified 6 genes mapping to a credible variant with PPA>5% that was deleteriously protein-relevant within the gene (CADD ≥15 [4]), 1 gene mapping to a >5% PPA credible variant that was an eQTL in kidney tissue [5,6], and 15 genes known for kidney-related phenotypes in human (OMIM [7], or Groopman et al. [1]). For presentation in the main manuscript (**Figure 7**), the list of genes with evidenced kidney phenotype was manually reviewed and reduced.

									99% credible variants (PPA > 0.05) within the gene with CADD ≥ 15			99% credible variants (PPA > 0.05) within the locus				Evidenced kidney phenotype for gene
												eQTL			sQTL	
									1	1	1	1	1	1	1	1
Locus name	Gene	distance to signal index variant	Chr of gene	Position start of gene	Position end of gene	credible variants in the signal	credible variants in the signal with PPA> 0.05	Score	stop-gained/ stop-lost/ non-synonymus	canonical-splice/ noncoding-change/ synonymous/ splice-site	other	NEPTUNE glomerulus	NEPTUNE tubulointerstitium	GTEx kidney	GTEx kidney	OMIM Human
[ALPL]	*CDC42*	473515	1	22379119	22419436	40	2	1	0	0	0	0	0	0	0	1
[ALPL]	*ALPL*	-699	1	21835857	21904905	40	2	1	0	0	0	0	0	0	0	2
[ALPL]	*HSPG2*	243120	1	22148724	22263790	40	2	1	0	0	0	0	0	0	0	1
[ARMC4]	*RAB18*	-411251	10	27793102	27831166	201	0	1	0	0	0	0	0	0	0	1
[AUTS2]	*AUTS2*	0	7	69063904	70258054	69	7	1	0	0	1	0	0	0	0	0
[CUBN]	*CUBN*	0	10	16865964	17171816	737	7	2	1	0	0	0	0	0	0	1
[DOCK7]	*KANK4*	-316040	1	62701836	62785083	225	0	1	0	0	0	0	0	0	0	1
[EEF1DP3]	*BRCA2*	390698	13	32889616	32973809	127	2	1	0	0	0	0	0	0	0	2
[FAT4]	*FAT4*	0	4	126237566	126414087	8	8	1	0	0	0	0	0	0	0	2
[LIMCH1]	*SLC30A9*	327736	4	41992522	42089551	62	5	1	0	0	0	0	0	0	0	1
[LOXL4]	*HPS1*	155382	10	100175954	100206704	110	2	1	0	0	0	0	0	0	0	1
[LOXL4]	*HPSE2*	196261	10	100216833	100995632	110	2	1	0	0	0	0	0	0	0	2
[MSC]	*EYA1*	-373623	8	72109667	72274467	11	4	1	0	0	0	0	0	0	0	2
[POLD3]	*C2CD3*	-479550	11	73723758	73882064	72	3	1	0	0	0	0	0	0	0	1
[RASSF6]	*RASSF6*	0	4	74437266	74486348	7	2	1	1	0	0	0	0	0	0	0
[REM2]	*SLC7A7*	-73240	14	23242431	23289020	140	4	1	0	0	0	0	0	0	0	2
[SLC2A2]	*TNIK*	68577	3	170780291	171178197	7	3	1	0	0	0	2	0	0	0	0
[SLC2A2]	*SLC2A2*	2422	3	170714136	170744768	7	3	1	0	0	0	0	0	0	0	2
[SLC2A4]	*DVL2*	-47916	17	7128660	7137863	56	7	1	1	0	0	0	0	0	0	0
[SLC2A4]	*SLC2A4*	0	17	7185053	7191367	56	7	1	0	0	1	0	0	0	0	0
[TET2]	*PPA2*	247206	4	106290233	106395227	48	7	1	0	0	0	0	0	0	0	1
[ZFP36L1]	*ZFP36L1*	-17198	14	69254371	69262960	32	5	1	0	0	1	0	0	0	0	0

Supplementary Note 1. Details on stage 1 overall+difference test approach.

Our stage 1 data (CKDGen and UKB) has been analyzed before for eGFR without DM-/noDM-stratification as described previously [2]. By this, we yielded 634 independent genome-wide significant variant associations ($P_{Overall}$<5x10^{-8}). This was achieved by a step-wise forward selection using approximate conditional analyses: independent variants in a larger region were identified by conditioning on the iteratively identified variants starting by conditioning on the variant with the smallest P-value. Among these 634 variants, 611 variants were analyzable also in DM and noDM, separately. The other 23 variants did not pass quality control filtering thresholds in DM or noDM requiring at least 36 studies in the meta-analysis per stratum. The rs79924715 variant was also excluded because it had already been identified by the difference test approach (P_{Diff}<5x10^{-8}). The second variant identified by the difference test approach, rs1223328 near *PDE9A*, had a $P_{Overall}$>=5x10^{-8} and was thus not among the 634 variants. Taken together, we took 610 variants with $P_{Overall}$<5x10^{-8} forward to the difference test.

Among the 610 variants, there were three independent variants in the *UMOD-PDILT* locus besides rs79924715, but only one of these, rs34882080, was significant at P_{Diff}< 0.05/610=8.2x10^{-5}, **Supplementary Data 2**). When evaluating the independence of the difference association for rs34882080 by conditioning on rs79924715 via GCTA, we found its difference association to disappear, but the rs7992415 difference association to remain (**Supplementary Figure 2**).

Supplementary Note 2. Robustness of the observed differences.

To investigate the robustness of the seven identified DM/noDM-differential eGFR associations, we performed sensitivity analyses using data from UKB and MVP.

First, we evaluated whether the observed patterns of difference were consistent also when eGFR was analyzed on the original scale instead of log-transformed scale [8]. When using combined data from UKB and MVP (n_{DM}=77,047, n_{nodM}=539,930) and eGFR without log-transformation, we observed consistent patterns of differences for all identified variants (**Supplementary Table 1**).

Second, we used a SNP-by-DM interaction term in the regression model (instead of the difference test based on DM-stratified effects sizes in the main analysis) based on linear regression and unrelated individuals of European ancestry in UKB (n_{DM}=19,277, n_{noDM}=348,728). We observed significant interaction effects that were directionally consistent with the difference observed by the stratified approach (**Supplementary Data 4**).

Third, interactions with other correlated covariates unaccounted for in the SNP-by-DM interaction analyses are known potential sources of bias in gene-environment interaction studies [9]. Therefore, we explored extended interaction models using again the unrelated UKB participants of European ancestry (n_{DM}=19,277, n_{noDM}=348,728): the SNP-by-DM interaction

associations were stable also after accounting for SNP-by-Age, SNP-by-Sex, SNP-by-Hypertension or SNP-by-BMI interaction (**Supplementary Data 4**).

In summary, our sensitivity analyses supported the robustness of our observed significant DM/noDM-differential effects at the seven identified loci.

Supplementary Note 3. Power considerations.

We conducted power computations to evaluate the minimum interaction effect size detectable by our combined stage 1+2 data with 180,000 individuals with DM and 1,300,000 individuals with noDM. For this, we considered log-transformed eGFR (age-/sex-adjusted residuals) and a standard deviation as observed in UKB (sd_{DM}=0.21 log(ml/min/1.73m), sd_{noDM}=0.15 log(ml/min/1.73m²)). We calculated power analytically [10] to identify DM/noDM-difference by the difference approach ($P_{Diff}<5x10^{-8}$) or by the overall+difference approach ($P_{Overall}<5x10^{-8}$ and $P_{Diff}<0.05/610$, assuming 610 overall associated variants). We considered different effect sizes on log eGFR in DM and noDM based on realistic effects sizes. In UKB, effect sizes on log eGFR overall of the 424 Stanzick *et al* [2] lead variants ranged from 0.0008 log(ml/min/1.73m²) (small) to 0.013 log(ml/min/1.73m²) (large); the *UMOD/PDILT* lead variant showed a large effect size of 0.011 log(ml/min/1.73m²). The minor allele frequencies (MAF) of these variants ranged from 1% to 50% with a median of 30%.

We first focused on a common variant (MAF=30%) and calculated power for varying log eGFR effect sizes in DM and noDM (**Supplementary Figure 6a**). For example, assuming a large genetic effect on eGFR of 0.011 log(ml/min/1.73m²) (comparable to the *UMOD/PDILT* variant), we had >99% power to identify an interaction when the large effect is noDM- or DM-only (**Supplementary Figure 6a,d**). Considering reduced effect sizes of 0.004 log(ml/min/1.73m²) (medium effect), or 0.001 log(ml/min/1.73m²) (small effect), the power to identify the interaction was (i) 92% and <1%, respectively for a medium and small noDM-only effect and (ii) 35% and <1%, respectively for a medium and small DM-only effect (**Supplementary Figure 6a,d**). For less frequent variants (MAF<=5%), power was limited (**Supplementary Figure 6b-d**).

The minimum interaction effect size detectable at 80% power for a common variant (MAF=30%) was (i) 0.0037 log(ml/min/1.73m²) for a noDM-only effect and (ii) 0.0050 log(ml/min/1.73m²) for a DM-only effect. For low frequency variants (MAF=5%), the minimum detectable effect sizes were (i) 0.0077 log(ml/min/1.73m²) for a noDM-only effect and (ii) 0.011 log(ml/min/1.73m²) for a DM-only effect (**Supplementary Figure 6b,d**).

In summary, the power of the two approaches to detect DM/noDM-differences in genetic effects on eGFR was high for common variants with large noDM-only and DM-only effects as well as for medium noDM-only effects; but limited for medium DM-only effects. For

less frequent or rare variants, realistic DM/noDM-differences in genetic effects on eGFR were not detectable.

Supplementary Note 4. Extended study acknowledgments.

**AA-DHS**	NIH R01 DK071891 (Barry I. Freedman, PI); NIH R01 NS075107 (Barry I. Freedman, PI).
**ADVANCE**	ADVANCE genomic sub-studies were supported by grants from the Ministry of Science and Innovation from the Quebec Government, from Genome Quebec, from the Consortium Québécois du Médicament, from the Canadian Institutes of Health Research and from Medpharmgene, OPTITHERA Inc and Les Laboratoires Servier.
**AFTER EU**	The AFTER EU study is the Danish part of the EURAGEDIC study which was supported by the European Commission (contract QLG2-CT-2001– 01669). The genotyping for this study was part of the Genetics of Diabetic Nephropathy (GenDN) study, primarily funded by Juvenile Diabetes Research Foundation (JDRF) International Prime Award Number 17-2013- 8. Tarunveer S Ahluwalia was also funded by the GenDN study grant and Lundbeck foundation Travel Grant (Ref. Number 2013-14471).
**AGES**	This study has been funded by NIA contract N01-AG-12100 and HHSN271201200022C with contributions from NEI, NIDCD and NHLBI, the NIA Intramural Research Program, Hjartavernd (the Icelandic Heart Association), and the Althingi (the Icelandic Parliament). The study is approved by the Icelandic National Bioethics Committee, VSN: 00-063. The researchers thank the participants for their willingness to participate in the study.
**Airwave**	We thank all participants in the Airwave Health Monitoring Study. The Airwave Health Monitoring Study is funded by the UK Home Office (780- TETRA; 2003-2018) andthe UK Medical Research Council and Economic & Social Research Council (MR/R023484/1; 2018-) with additional support from the National Institute for Health Research(NIHR Imperial College Biomedical Research Centre in collaboration with Imperial College NHS Healthcare Trust. The views expressed are those of the authors and not necessarily those of the sponsors. PE acknowledges support from the Medical Research Council(MR/L01341X/1; MR/S019669/1) for the MRC Centre for Environment and Health. This work used computing resources of the UK MEDical BIOinformatics partnership (UK MED-BIO supported by the Medical Research Council (MR/L01632X/1).PE is supported by the UK Dementia Research

Institute funded by the UK Medical Research Council, Alzheimer's Society and Alzheimer's Research UK (MC_PC_17114) and the Imperial College BritishHeart Foundation Centre for Research Excellence (RE/18/4/34215). PE is associate director of the Health Data Research UK London funded by a consortium led by the UK Medical Research Council.

Amish We thank the Amish research volunteers for their long-standing partnership in research, and the research staff at the Amish Research Clinic for their work and dedication. The Amish contribution was supported by NIH grants R01 AG18728, R01 HL088119, U01 GM074518, U01 HL072515, U01 HL084756, and NIH K12RR023250, and P30 DK072488. Additional support was provided by the University of Maryland General Clinical Research Center, grant M01 RR 16500, the Baltimore Veterans Administration Medical Center Geriatrics Research, and the Paul Beeson Physician Faculty Scholars in Aging Program.

ARIC The Atherosclerosis Risk in Communities study has been funded in whole or in part with Federal funds from the National Heart, Lung, and Blood Institute, National Institutes of Health, Department of Health and Human Services (contract numbers HHSN268201700001I, HHSN268201700002I, HHSN268201700003I, HHSN268201700004I and HHSN268201700005I), R01HL087641, R01HL086694; National Human Genome Research Institute contract U01HG004402; and National Institutes of Health contract HHSN268200625226C. The work of AK was funded by the German Research Foundation (DFG) KO 3598/5-1. The authors thank the staff and participants of the ARIC study for their important contributions. Infrastructure was partly supported by Grant Number UL1RR025005, a component of the National Institutes of Health and NIH Roadmap for Medical Research.

ASPS-Fam The authors thank the staff and participants of the ARIC study for their important contributions. Infrastructure was partly supported by Grant Number UL1RR025005, a component of the National Institutes of Health and NIH Roadmap for Medical Research. The work of Anna Köttgen was supported by a Heisenberg Professorship (KO 3598/3- 1) as well as CRCs 1140 and 992 of the German Research Foundation. The authors thank the staff and the participants for their valuable contributions. We thank Birgit Reinhart for her long-term administrative commitment, Elfi Hofer for the technical assistance at creating the DNA bank, Ing. Johann Semmler and Anita Harb for DNA sequencing and DNA analyses by TaqMan assays and Irmgard Poelzl for supervising the quality management processes after ISO9001 at the

biobanking and DNA analyses.The research reported in this article was funded by the Austrian Science Fond (FWF) grant number P20545-P05, P13180 and PI904 as well as by the Austrian National Bank (OeNB) Anniversary Fund grant number P15435 and the Austrain Federal Ministry of Science, Research and Economy under the aegis of the EU Joint ProgrammeNeurodegenerative Disease Research (JPND)-www.jpnd.eu. The Medical University of Graz supports the databank of the ASPS.

BBJ The BioBank Japan project is supported by the Ministry of Education, Culture, Sports, Sciences and Technology of Japanese government and the Japan Agency for Medical Research and Development. Y.O. was supported by JSPS KAKENHI (19H01021, 20K21834), and AMED (JP21km0405211, JP21ek0109413, JP21ek0410075, JP21gm4010006, and JP21km0405217), JST Moonshot R&D (JPMJMS2021, JPMJMS2024), Takeda Science Foundation.

BES BES was supported by the National Natural Science Foundation of China, (81570835), and the Beijing Municipal of Health Development Project (2019-4).

BioMe The Mount Sinai IPM Biobank Program is supported by The Andrea and Charles Bronfman Philanthropies. Ruth Loos is funded by R01DK110113, U01HG007417, R01DK101855, and R01DK107786.

CHNS The China Health and Nutrition Survey (CHNS) was supported by the China National Institute of Nutrition and Food Safety; the China Center for Disease Control; the National Institutes of Health (R01HD30880, R01HL108427, and R01DK104371); the Fogarty International Center of the National Institutes of Health; the China-Japan Friendship Hospital; the Chinese Ministry of Health; and the Carolina Population Center (R24 HD050924). Cassandra N. Spracklen was supported by the American Heart Association Postdoctoral Fellowship (17POST3650016). Karen Mohlke was supported by R01DK072193 and U01DK105561.

CHRIS CHRIS study investigators thank all study participants, all general practitioners, the personnel of the Hospital of Silandro/Schlanders, and the personnel of the South Tyrolean Healthcare System for their participation and collaboration in the project. Full acknowledgements for the CHRIS study are reported at https://translational-medicine.biomedcentral.com/articles/10.1186/s12967-015-0704-9. The CHRIS study was funded by the Department of Innovation, Research, and University of the Autonomous Province of Bolzano/Bozen.

CHS Cardiovascular Health Study: This CHS research was supported by NHLBI contracts HHSN268201200036C, HHSN268200800007C, HHSN268201800001C, N01HC55222, N01HC85079, N01HC85080, N01HC85081, N01HC85082, N01HC85083, N01HC85086; and NHLBI grants U01HL080295, R01HL087652, R01HL105756, R01HL103612, R01HL120393, U01HL130114, HL130114 and 75N92021D00006. with additional contribution from the National Institute of Neurological Disorders and Stroke (NINDS). Additional support was provided through R01AG023629 from the National Institute on Aging (NIA). A full list of principal CHS investigators and institutions can be found at CHS-NHLBI.org. The provision of genotyping data was supported in part by the National Center for Advancing Translational Sciences, CTSI grant UL1TR001881, and the National Institute of Diabetes and Digestive and Kidney Disease Diabetes Research Center (DRC) grant DK063491 to the Southern California Diabetes Endocrinology Research Center. The content is solely the responsibility of the authors and does not necessarily represent the official views of the National Institutes of Health.

Cilento We thank the populations of Cilento for their participation in the study. This work was supported by grants from the Italian Ministry of Universities (PON03PE_00060_7, IDF SHARID ARS01_01270), the Assessorato Ricerca Regione Campania.

CoLaus The CoLaus|PsyCoLaus study was and is supported by research grants from GlaxoSmithKline, the Faculty of Biology and Medicine of Lausanne, and the Swiss National Science Foundation (grants 3200B0–105993, 3200B0-118308, 33CSCO-122661, 33CS30-139468 and 33CS30-148401).

CROATIA-Korcula, CROATIA-Split, CROATIA-Vis 10001 Dalmatians: The Croatian Biobank (CROATIA) The CROATIA-Vis, CROATIA-Korcula and CROATIA-Split were funded by grants from the Medical Research Council (UK), from the Republic of Croatia Ministry of Science, Education and Sports (108-1080315-0302; 216-1080315-0302) and the Croatian Science Foundation (8875); and the CROATIA-Korčula genotyping was funded by the European Union framework program 6 project EUROSPAN (LSHGCT2006018947). We thank the staff of several institutions in Croatia that supported the field work, including Zagreb Medical Schools, the Institute for Anthropological Research in Zagreb, the recruitment team from the Croatian Centre for Global Health, University of Split and all the study participants. We are grateful to the Helmholtz Zentrum Munchen (Munich, Germany), AROS Applied Biotechnology, (Aarhus, Denmark) and

the Edinburgh Clinical Research facility, University of Edinburgh (Edinburgh, United Kingdom) for SNP array genotyping. Genetic analyses were supported by the MRC HGU “QTL in Health and Disease” (MRC University Unit Programme Grant MC_UU_00007/10).

DECODE The study was funded by deCODE Genetics/Amgen inc. We thank the study subjects for their valuable participation and our colleagues, who contributed to data collection, sample handling, and genotyping.

DIACORE Cohort recruiting and management was funded by the KfH Stiftung Präventivmedizin e.V. (Carsten A. Böger). Genome-wide genotyping was funded the Else Kröner-Fresenius-Stiftung (2012_A147), the KfH Stiftung Präventivmedizin and the University Hospital Regensburg. The Deutsche Forschungsgemeinschaft (DFG, German Research Foundation) supported this work – Project-ID 387509280 – SFB 1350 (Subproject C6 to I.M.H.) and Iris Heid and Carsten Böger received funding by DFG BO 3815/4-1.

Diabetic Cohort The Diabetic Cohort (DC) was supported by the individual research grant from the National Medical Research Council (NMRC) and the Biomedical Research Council (BMRC) of Singapore. The Genome Institute of Singapore provided services for genotyping.

EGCUT The EGCUT studies were financed by Estonian Government (grants IUT20-60 and IUT24-6) and by European Commission through the European Regional Development Fund in the frame of grant Estonian Center of Genomics/Roadmap II (project No. 2014-2020.4.01.16-0125) and grant GENTRANSMED (Project No. 2014-2020.4.01.15-0012) and through H2020 grant no 692145 (ePerMed).
This study was supported by the Estonian Research Council grant PRG687. Data analyses were carried out in part in the High-Performance Computing Center of University of Tartu. We thank participants and support staff of Estonian Biobank.

ESTHER The ESTHER study was funded by the Baden-Württemberg state Ministry of Science, Research and Arts (Stuttgart, Germany), the Federal Ministry of Education and Research (Berlin, Germany), and the Federal Ministry of Family Affairs, Senior Citizens, Women and Youth (Berlin, Germany).

FamHS The study was supported by grant R01-DK-089256 from NIDDK and grant R01HL117078 from NHLBI.

FHS The Framingham Heart Study is supported by HHSN268201500001.

FINCAVAS The Finnish Cardiovascular Study (FINCAVAS) has been financially supported by the Competitive Research Funding of the Tampere University

Hospital (Grant 9M048 and 9N035), the Finnish Cultural Foundation, the Finnish Foundation for Cardiovascular Research, the Emil Aaltonen Foundation, Finland, the Tampere Tuberculosis Foundation, EU Horizon 2020 (grant 755320 for TAXINOMISIS and grant 848146 for To Aition), and the Academy of Finland grant 322098. The authors thank the staff of the Department of Clinical Physiology for collecting the exercise test data.

Finrisk We thank all individuals who participated in Finrisk surveys. Veikko Salomaa, Markus Perola and Mikko Kuokkanen were supported by the Finnish Foundation for Cardiovascular Research. Mikko Kuokkanen was also supported by Päivikki and Sakari Sohlberg Foundation and Yrjö Jahnsson Foundation. We thank the CSC – IT center for science, Finland, for providing computational resources.

GCKD The GCKD study was funded by the German Ministry of Research and Education (Bundesminsterium für Bildung und Forschung, BMBF), by the Foundation KfH Stiftung Präventivmedizin and by the Deutsche Forschungsgemeinschaft (DFG, German Research Foundation) – Projektnummer 246781735 – SFB 1140. Unregistered grants to support the study were provided by Bayer, Fresenius Medical Care and Amgen. Genotyping was supported by Bayer Pharma AG. Uromodulin measurements in GCKD were supported by the Swiss National Centre of Competence in Research Kidney Control of Homeostasis program and the Swiss National Science Foundation grant 31003A_169850. The work of Matthias Wuttke was supported by DFG CRC 1140 and the Else Kroener Fresenius Forschungskolleg NAKSYS. The work of Yong Li was supported by DFG KO 3598/4-1. The work of Anselm Hoppmann was partly supported by NIDDK R01 DK087635-09.

Generation R The Generation R Study is conducted by the Erasmus Medical Center in close collaboration with the School of Law and Faculty of Social Sciences of the Erasmus University Rotterdam, the Municipal Health Service Rotterdam area, Rotterdam, the Rotterdam Homecare Foundation, Rotterdam and the Stichting Trombosedienst & Artsenlaboratorium Rijnmond (STAR-MDC), Rotterdam. We gratefully acknowledge the contribution of children and parents, general practitioners, hospitals, midwives and pharmacies in Rotterdam. The generation and management of GWAS genotype data for the Generation R Study were done at the Genetic Laboratory of the Department of Internal Medicine, Erasmus MC, The Netherlands. We would like to thank Karol Estrada, Dr. Tobias A. Knoch, Anis Abuseiris, Luc V. de Zeeuw, and

Rob de Graaf, for their help in creating GRIMP, BigGRID, MediGRID, and Services@MediGRID/D-Grid, (funded by the German Bundesministerium fuer Forschung und Technology; grants 01 AK 803 A-H, 01 IG 07015 G) for access to their grid computing resources. We thank Mila Jhamai, Manoushka Ganesh, Pascal Arp, Marijn Verkerk, Lizbeth Herrera and Marjolein Peters for their help in creating, managing and QC of the GWAS database. Also, we thank Karol Estrada for their support in creation and analysis of imputed data.The general design of Generation R Study is made possible by financial support from the Erasmus Medical Center, Rotterdam, the Erasmus University Rotterdam, the Netherlands Organization for Health Research and Development (ZonMw), the Netherlands Organisation for Scientific Research (NWO), the Ministry of Health, Welfare and Sport and the Ministry of Youth and Families. This project received funding from the European Union's Horizon 2020 Research and Innovation Programme under grant agreements 733206 (LifeCycle), 874739 (LongITools) and 824989, (EUCAN-Connect). Fernando Rivadeneira received additional funding from the Netherlands Organization for Health Research and Development (VIDI 016. 136. 367).

GS:SFHS Generation Scotland received core support from the Chief Scientist Office of the Scottish Government Health Directorates [CZD/16/6] and the Scottish Funding Council [HR03006]. We thank all the families who took part, the general practitioners and the Scottish School of Primary Care for their help in recruiting them, and the whole Generation Scotland team, which includes interviewers, computer and laboratory technicians, clerical workers, research scientists, volunteers, managers, receptionists, healthcare assistants and nurses. Genotyping of the GS:SFHS samples was carried out by the Genetics Core Laboratory at the Edinburgh Clinical Research Facility, University of Edinburgh, Scotland and was funded by the Medical Research Council UK and the Wellcome Trust (Wellcome Trust Strategic Award "STratifying Resilience and Depression Longitudinally" (STRADL) Reference 104036/Z/14/Z.

GSK Funding Source: Max-Planck Society, German Federal Ministry of Education and Research (BMBF) in the framework of the National Genome Research Network (NGFN), Foerderkennzeichen 01GS0481.

HANDLS The authors thank Dr. Salman M. Tajuddin, all study participants and the Healthy Aging in Neighborhoods of Diversity across the Life Span (HANDLS) study medical staff for their contributions. The HANDLS study was supported

by the Intramural Research Program of the NIH, National Institute on Aging and the National Center on Minority Health and Health Disparities (project # Z01-AG000513 and human subjects protocol number 09-AG-N248). Data analyses for the HANDLS study utilized the high-performance computational resources of the Biowulf Linux cluster at the National Institutes of Health, Bethesda, MD. (http://biowulf.nih.gov; http://hpc.nih.gov).

**HUNT**	The Trøndelag Health Study (The HUNT Study) is a collaboration between HUNT Research Centre (Faculty of Medicine and Health Sciences, NTNU, Norwegian University of Science and Technology), Trøndelag County Council, Central Norway Regional Health Authority, and the Norwegian Institute of Public Health. The HUNT study was approved by the Central Norway Regional Committee for Medical and Health Research Ethics (REC Central no. 2015/1188), and written informed consent was given by all participants.
**HYPERGENES**	Funding Source: HYPERGENES project (FP7-HEALTH-F4-2007-201550) and InterOmics (PB05 MIUR-CNR Italian Flagship Project).
**INGI-CARL**	The project was approved by the local administration of Carlantino, the Health Service of Foggia Province, Italy, and ethical committee of the IRCCS Burlo-Garofolo of Trieste. We thank the people of Carlantino for their support.
**INGI-FVG**	Project co-financed by the European Regional Development Fund under the Regional Operational Programme of Friuli Venezia Giulia - Objective "Regional Competitiveness and Employment" 2007/2013, Telethon Foundation (GGP09037), Fondo Trieste (2008), Regione FVG (L.26.2008), and Italian Ministry of Health (RC16/06, ART. 13 D.LGS 297/99) (to Paolo Gasparini). We thank the people of Friuli Venezia Giulia Region for their support.
**INGI-VBI**	The research was supported by funds from Compagnia di San Paolo, Torino, Italy; Fondazione Cariplo, Italy and Ministry of Health, Ricerca Finalizzata 2011 and CCM 2010 to Daniela Toniolo. The funders had no role in study design, data collection and analysis, decision to publish, or preparation of the manuscript. We thank the inhabitants of the VB that made this study possible, the local administrations, the Tortona and Genova archdiocese and the ASL-22, Novi Ligure (Al) for support. We thank Clara Camaschella for data collection supervision and organization of the clinical data collection, Fiammetta Viganò for technical help, and Corrado Masciullo for the analysis platform.

JHS The Jackson Heart Study (JHS) is supported and conducted in collaboration with Jackson State University (HHSN268201800013I), Tougaloo College (HHSN268201800014I), the Mississippi State Department of Health (HHSN268201800015I/HHSN26800001) and the University of Mississippi Medical Center (HHSN268201800010I, HHSN268201800011I and HHSN268201800012I) contracts from the National Heart, Lung, and Blood Institute (NHLBI) and the National Institute for Minority Health and Health Disparities (NIMHD). The authors also wish to thank the staffs and participants of the JHS. Laura M. Raffield is supported by T32 HL129982.

JUPITER The JUPITER trial and its genetic substudy were funded by AstraZeneca.

KORA The KORA research platform (KORA, Cooperative Health Research in the Region of Augsburg) was initiated and financed by the Helmholtz Zentrum München - German Research Center for Environmental Health, which is funded by the German Federal Ministry of Education and Research and by the State of Bavaria. Furthermore, KORA research was supported within the Munich Center of Health Sciences (MC Health), Ludwig-Maximilians Universität, as part of LMUinnovativ. Statistical KORA analyses were supported by DFG BO-3815/4-1 (to Carsten A. Böger), BMBF 01ER1206, 01ER1507 (to Iris M. Heid), by the University of Regensburg and by the Deutsche Forschungsgemeinschaft (DFG, German Research Foundation) – Project-ID 387509280 – SFB 1350 (Subproject C6 to I.M.H.) and DFG BO 3815/4-1.

LIFE-Adult, LIFE-Heart, LIFE-Child LIFE-Adult, LIFE-Heart, and LIFE-Child, are funded by the Leipzig Research Center for Civilization Diseases (LIFE). LIFE is an organizational unit affiliated to the Medical Faculty of the University of Leipzig. LIFE is funded by means of the European Union, by the European Regional Development Fund (ERDF) and by funds of the Free State of Saxony within the framework of the excellence initiative.

Lifelines The LifeLines Cohort Study, and generation and management of GWAS genotype data for the LifeLines Cohort Study is supported by the UMCG Genetics Lifelines Initiative (UGLI), the Netherlands Organization of Scientific Research NWO (grant 175.010.2007.006), the Economic Structure Enhancing Fund (FES) of the Dutch government, the Ministry of Economic Affairs, the Ministry of Education, Culture and Science, the Ministry for Health, Welfare and Sports, the Northern Netherlands Collaboration of Provinces (SNN), the Province of Groningen, University Medical Center Groningen, the University of Groningen, Dutch Kidney Foundation and Dutch Diabetes Research

Foundation. The authors wish to acknowledge the services of the Lifelines Cohort Study, the contributing research centers delivering data to Lifelines, and all the study participants.

**Living-Biobank**	The genotyping was funded by the Agency for Science, Technology and Research, Singapore (https://www.a-star.edu.sg/), and by Merck Sharp & Dohme Corp., Whitehouse Station, NJ USA (http://www.merck.com). The participants were from the MEC study (funded by the Biomedical Research Council (BMRC 03/1/27/18/216), National Medical Research Council (0838/2004), National Research Foundation (through BMRC 05/1/21/19/425 and 11/1/21/19/678), Ministry of Health, Singapore, National University of Singapore and National University Health System, Singapore), and The SH2012 study (funded by the Ministry of Health, Singapore, National University of Singapore and National University Health System, Singapore).
**LLFS**	The study was supported by grants: U01AG023746, U01AG023712, U01AG023749, U01AG023755, U01AG023744, and U19AG063893 from NIA.
**LOLIPOP**	The LOLIPOP study is supported by the National Institute for Health Research (NIHR) Comprehensive Biomedical Research Centre Imperial College Healthcare NHS Trust, the British Heart Foundation (SP/04/002), the Medical Research Council (G0601966, G0700931), the Wellcome Trust (084723/Z/08/Z, 090532 & 098381) the NIHR (RP-PG-0407-10371), the NIHR Official Development Assistance (ODA, award 16/136/68), the European Union FP7 (EpiMigrant, 279143), and H2020 programs (iHealthT2D, 643774). We acknowledge support of the MRC-PHE Centre for Environment and Health, and the NIHR Health Protection Research Unit on Health Impact of Environmental Hazards. The work was carried out in part at the NIHR/Wellcome Trust Imperial Clinical Research Facility. The views expressed are those of the author(s) and not necessarily those of the Imperial College Healthcare NHS Trust, the NHS, the NIHR or the Department of Health. We thank the participants and research staff who made the study possible. JC is supported by the Singapore Ministry of Health’s National Medical Research Council under its Singapore Translational Research Investigator (STaR) Award (NMRC/STaR/0028/2017).
**LURIC**	LURIC was supported by the 7th Framework Program of the EU (AtheroRemo, grant agreement number 201668 and RiskyCAD, grant agreement number 305739). The work of G.E.D. is supported by the European Union’s Horizon 2020 research and innovation programme under

the ERA-Net Cofund action N° 727565 (OCTOPUS project) and the German Ministry of Education and Research (grant number 01EA1801A). The authors thank the LURIC study team involved in patient recruitment as well as sample and data handling, and the laboratory staff at the Ludwigshafen General Hospital and the universities of Freiburg, Ulm and Heidelberg, Germany.

MDC-CC This study was supported by the European Research Council (Consolidator grant nr 649021, Orho-Melander), the Swedish Research Council, the Swedish Heart and Lung Foundation, the Novo Nordic Foundation, the Swedish Diabetes Foundation, and the Påhlsson Foundation, and by equipment grants from the Knut and Alice Wallenberg Foundation, the Region Skåne, Skåne University Hospital, the Linneus Foundation for the Lund University Diabetes Center and Swedish Foundation for Strategic Research for IRC15-0067.

MESA MESA and the MESA SHARe projects are conducted and supported by the National Heart, Lung, and Blood Institute (NHLBI) in collaboration with MESA investigators. Support for MESA is provided by contracts 75N92020D00001, HHSN268201500003I, N01-HC-95159, 75N92020D00005, N01-HC-95160, 75N92020D00002, N01-HC-95161, 75N92020D00003, N01-HC-95162, 75N92020D00006, N01-HC-95163, 75N92020D00004, N01-HC-95164, 75N92020D00007, N01-HC-95165, N01-HC-95166, N01-HC-95167, N01-HC-95168, N01-HC-95169, UL1-TR-000040, UL1-TR-001079, UL1-TR-001420. Funding for SHARe genotyping was provided by NHLBI Contract N02-HL-64278. Genotyping was performed at Affymetrix (Santa Clara, California, USA) and the Broad Institute of Harvard and MIT (Boston, Massachusetts, USA) using the Affymetrix Genome-Wide Human SNP Array 6.0. MESA Family is conducted and supported by the National Heart, Lung, and Blood Institute (NHLBI) in collaboration with MESA investigators. Support is provided by grants and contracts R01HL071051, R01HL071205, R01HL071250, R01HL071251, R01HL071258, R01HL071259, by the National Center for Research Resources, Grant UL1RR033176. This publication was developed under Science to Achieve Results (STAR) research assistance agreements, No. RD831697 (MESA Air), and RD-83830001 (MESA Air Next Stage), awarded by the U.S Environmental protection Agency. It has not been formally reviewed by the EPA. The views expressed in this document are solely those of the authors and the EPA does not endorse any products or commercial services mentioned in this publication. The provision of genotyping data was supported in part by the

National Center for Advancing Translational Sciences, CTSI grant UL1TR001881, and the National Institute of Diabetes and Digestive and Kidney Disease Diabetes Research Center (DRC) grant DK063491 to the Southern California Diabetes Endocrinology Research Center. Infrastructure for the CHARGE Consortium is supported in part by the National Heart, Lung, and Blood Institute (NHLBI) grant R01HL105756.

METSIM Michael Boehnke was supported by NIH grant DK062370. Markku Laakso was supported by the Academy of Finland (grant no. 321428) and the Sigrid Juselius Foundation.

MGI The authors acknowledge the Michigan Genomics Initiative participants, Precision Health at the University of Michigan, the University of Michigan Medical School Central Biorepository, and the University of Michigan Advanced Genomics Core for providing data and specimen storage, management, processing, and distribution services, and the Center for Statistical Genetics in the Department of Biostatistics at the School of Public Health for genotype data curation, imputation, and management in support of the research reported in this publication.

MICROS MICROS study investigators thank all study participants, all primary care practitioners, and the personnel of the Hospital of Silandro/Schlanders, and the personnel of the South Tyrolean Health System for their participation and collaboration in the project.. The study was supported by the Ministry of Health and Department of Educational Assistance, University and Research of the Autonomous Province of Bolzano, the South Tyrolean Sparkasse Foundation, and the European Union framework program 6 EUROSPAN project (contract no. LSHG-CT- 2006-018947).

MyCode (Geisinger) We would like to acknowledge the participants, staff, and our colleagues associated with the Geisinger MyCode Community Health Initiative. We also thank the staff of the PACDC of Geisinger for assistance with the phenotypic data, and the staff of the Biomedical & Translational Informatics and Kidney Health Research Institute.

MVP The work on MVP was funded by the Veterans Health Administration Clinical Science & Research MeritCX00189. “Genetics of CKD and Hypertension-Risk Prediction and Drug Response” in the MVP to Adriana M. Hung.

NEO The authors of the NEO study thank all individuals who participated in the Netherlands Epidemiology in Obesity study, all participating general practitioners for inviting eligible participants and all research nurses for collection of the data. We thank the NEO study group, Pat van Beelen, Petra

Noordijk and Ingeborg de Jonge for the coordination, lab and data management of the NEO study. The genotyping in the NEO study was supported by the Centre National de Génotypage (Paris, France), headed by Jean-Francois Deleuze. The NEO study is supported by the participating Departments, the Division and the Board of Directors of the Leiden University Medical Center, and by the Leiden University, Research Profile Area Vascular and Regenerative Medicine. Dennis Mook-Kanamori is supported by Dutch Science Organization (ZonMW-VENI Grant 916.14.023).

NESDA For NESDA, funding was obtained from the Netherlands Organization for Scientific Research (Geestkracht program grant 10-000-1002); the Center for Medical Systems Biology (CSMB, NWO Genomics), Biobanking and Biomolecular Resources Research Infrastructure (BBMRI-NL), VU University's Institutes for Health and Care Research (EMGO+) and Neuroscience Campus Amsterdam, University Medical Center Groningen, Leiden University Medical Center, National Institutes of Health (NIH, R01D0042157-01A, MH081802, Grand Opportunity grants 1RC2 MH089951 and 1RC2 MH089995). Part of the genotyping and analyses were funded by the Genetic Association Information Network (GAIN) of the Foundation for the National Institutes of Health. Computing was supported by BiG Grid, the Dutch e-Science Grid, which is financially supported by NWO.

OGP The Ogliastra Genetic Park study was supported by grant from the Italian Ministry of Education, University and Research (MIUR) no. 5571/DSPAR/2002. We thank all study participants for their contributions and the municipal administrations for their economic and logistic support.

ORCADES The Orkney Complex Disease Study (ORCADES) was supported by the Chief Scientist Office of the Scottish Government (CZB/4/276, CZB/4/710), a Royal Society URF to James F. Wilson, the MRC Human Genetics Unit quinquennial programme "QTL in Health and Disease", Arthritis Research UK and the European Union framework program 6 EUROSPAN project (contract no. LSHG-CT-2006-018947). DNA extractions were performed at the Wellcome Trust Clinical Research Facility in Edinburgh. We would like to acknowledge the invaluable contributions of the research nurses in Orkney, the administrative team in Edinburgh, the people of Orkney, and the data analysts in particular Dr Thibaud Boutin for the genotype imputation to the HRC reference panel.

PIVUS Prospective Investigation of the Vasculature in Uppsala Seniors **(PIVUS)** was supported by Wellcome Trust Grants (WT098017, WT064890, WT090532),

Uppsala University, Uppsala University Hospital, the Swedish Research Council and the Swedish Heart-Lung Foundation.

PREVEND The Prevention of Renal and Vascular Endstage Disease Study (PREVEND) genetics is supported by the Dutch Kidney Foundation (Grant E033), the EU project grant GENECURE (FP-6 LSHM CT 2006 037697), the National Institutes of Health (grant LM010098), the Netherlands organization for health research and development (NWO VENI grant 916.761.70), and the Dutch Inter University Cardiology Institute Netherlands (ICIN). Niek Verweij was supported by NWO VENI grant 016.186.125.

POPGEN The PopGen 2.0 network was supported by a grant from the German Ministry for Education and Research (01EY1103). AF and WL receive funding from the DFG Excellence Cluster “Precision Medicine in Chronic Inflammation” (PMI; EXC 2167)

QIMR adolescent We acknowledge funding by Australian National Health and Medical Research Council (NHMRC) grants 241944, 339462, 389927, 389875, 389891, 389892, 389938, 442915, 442981, 496739, 552485, 552498 and Australian Research Council grants A7960034, A79906588, A79801419, DP0770096, DP0212016, DP0343921 for building and maintaining the adolescent twin family resource through which samples were collected.

RS The Rotterdam Study (RS) has been approved by the Medical Ethics Committee of the Erasmus MC (registration number MEC 02.1015) and by the Dutch Ministry of Health, Welfare and Sport (Population Screening Act WBO, license number 1071272-159521-PG). The RS has been entered into the Netherlands National Trial Register (NTR; www.trialregister.nl) and into the WHO International Clinical Trials Registry Platform (ICTRP; www.who.int/ictrp/network/primary/en/) under shared catalogue number NTR6831. All participants provided written informed consent to participate in the study and to have their information obtained from treating physicians. The generation and management of GWAS genotype data for the RS (RS I, RS II, RS III) was executed by the Human Genotyping Facility of the Genetic Laboratory of the Department of Internal Medicine, Erasmus MC, Rotterdam, The Netherlands. The GWAS datasets are supported by the Netherlands Organisation of Scientific Research NWO Investments (nr. 175.010.2005.011, 911-03-012), the Genetic Laboratory of the Department of Internal Medicine, Erasmus MC, the Research Institute for Diseases in the Elderly (014-93-015; RIDE2), the Netherlands Genomics Initiative (NGI)/Netherlands Organisation for Scientific Research (NWO) Netherlands Consortium for Healthy Aging

(NCHA), project nr. 050-060- 810. We thank Pascal Arp, Mila Jhamai, Marijn Verkerk, Lizbeth Herrera and Marjolein Peters, MSc, and Carolina Medina-Gomez, MSc, for their help in creating the GWAS database, and Karol Estrada, PhD, Yurii Aulchenko, PhD, and Carolina Medina-Gomez, MSc, for the creation and analysis of imputed data. The RS is funded by Erasmus Medical Center and Erasmus University, Rotterdam, Netherlands Organization for the Health Research and Development (ZonMw), the Research Institute for Diseases in the Elderly (RIDE), the Ministry of Education, Culture and Science, the Ministry for Health, Welfare and Sports, the European Commission (DG XII), and the Municipality of Rotterdam. The authors are grateful to the study participants, the staff from the RS and the participating general practitioners and pharmacists.

**SCES**	The Singapore Chinese Eye Study (SCES) was supported by grants from the National Medical Research Council (STaR/0003/2008), the Singapore Bio Imaging Consortium (C-011/2006) and the Biomedical Research Council (08/1/35/19/550). The Genome Institute of Singapore provided services for genotyping.
**SCHS - CHD cases and controls**	The Singapore Chinese Health Study was supported by the National Institutes of Health, USA (RO1 CA144034 and UM1 CA182876), the nested case-control study of myocardial infarction by the Singapore National Medical Research Council (NMRC 1270/2010), and genotyping by the HUJ-CREATE Programme of the National Research Foundation, Singapore (Project Number 370062002).
**SHIP/SHIP-Trend**	SHIP is part of the Community Medicine Research net of the University of Greifswald, Germany, which is funded by the Federal Ministry of Education and Research (grants no. 01ZZ9603, 01ZZ0103, and 01ZZ0403), the Ministry of Cultural Affairs as well as the Social Ministry of the Federal State of Mecklenburg-West Pomerania, and the network ‘Greifswald Approach to Individualized Medicine (GANI_MED)’ funded by the Federal Ministry of Education and Research (grant 03IS2061A). Genome-wide data have been supported by the Federal Ministry of Education and Research (grant no. 03ZIK012) and a joint grant from Siemens Healthineers, Erlangen, Germany and the Federal State of Mecklenburg- West Pomerania. The University of Greifswald is a member of the Caché Campus program of the InterSystems GmbH.
**SiMES**	The Singapore Malay Eye Study (SiMES) was funded by the National Medical Research Council (NMRC 0796/2003 and NMRC/STaR/0003/2008) and

Biomedical Research Council (BMRC, 09/1/35/19/616). The Genome Institute of Singapore provided services for genotyping.

SINDI The Singapore Indian Eye Study (SINDI) was funded by grants from the Biomedical Research Council of Singapore (BMRC 09/1/35/19/616 and 08/1/35/19/550), and the National Medical Research Council of Singapore (NMRC/STaR/0003/2008). The Genome Institute of Singapore provided services for genotyping.

SKIPOGH The SKIPOGH study was supported by a Swiss national science foundation grant (FN33CM30-124087 and FN33CM30-140331), by the Swiss National Centres of Competence in Research Kidney and TransCure. Olivier Devuyst was supported by Swiss National Science Foundation (project grant 31003A-169850) and the Swiss National Centre of Competence in Research (NCCR) Kidney Control of Homeostasis (Kidney.CH).

SOLID-TIMI 52 The SOLID-TIMI 52 trial was supported and funded by grants from GlaxoSmithKline.

Sorbs This work was supported by grants from the German Research Foundation (SFB-1052 ‘‘Obesity mechanisms’’ A01, B03, SPP 1629 TO 718/2- 1), from the German Diabetes Association, from the DHFD (Diabetes Hilfs- und Forschungsfonds Deutschland) and from IFB Adiposity Diseases (AD2- 060E, AD2-06E95, AD2-06E99). IFB Adiposity Diseases is supported by the Federal Ministry of Education and Research (BMBF), Germany, FKZ:01EO1501.

SP2 The Singapore Prospective Study Program (SP2) were supported by the individual research grant and clinician scientist award schemes from the National Medical Research Council (NMRC) and the Biomedical Research Council (BMRC) of Singapore. The Genome Institute of Singapore provided services for genotyping.

STABILITY The STABILITY trial was supported and funded by grants from GlaxoSmithKline.

TRAILS TRAILS (TRacking Adolescents’ Individual Lives Survey) is a collaborative project involving various departments of the University Medical Center and University of Groningen, the University of Utrecht, the Radboud Medical Center Nijmegen, and the Parnassia Group, all in the Netherlands. TRAILS has been financially supported by various grants from the Netherlands Organization for Scientific Research NWO (Medical Research Council program grant GB-MW 940-38-011; ZonMW Brainpower grant 100-001-004; ZonMw Risk Behavior and Dependence grants 60-60600-97-118; ZonMw

Culture and Health grant 261-98-710; Social Sciences Council medium-sized investment grants GB-MaGW 480-01-006 and GB-MaGW 480-07-001; Social Sciences Council project grants GB-MaGW 452-04-314 and GB-MaGW 452-06-004; NWO large-sized investment grant 175.010.2003.005; NWO Longitudinal Survey and Panel Funding 481-08-013 and 481-11-001; NWO Vici 016.130.002 and 453-16-007/2735; NWO Gravitation 024.001.003), the Dutch Ministry of Justice (WODC), the European Science Foundation (EuroSTRESS project FP-006), the European Research Council (ERC-2017-STG-757364 en ERC-CoG-2015-681466), Biobanking and Biomolecular Resources Research Infrastructure BBMRI-NL (CP 32), the Gratama foundation, the Jan Dekker foundation, the participating universities, and Accare Centre for Child and Adolescent Psychiatry. Statistical analyses were carried out on the Genetic Cluster Computer (http://www.geneticcluster.org), which is financially supported by the Netherlands Scientific Organization (NWO 480-05-003) along with a supplement from the Dutch Brain Foundation. We are grateful to everyone who participated in this research or worked on this project to make it possible.

TwinGene TwinGene is a sub-study of the Swedish Twin Registry which is managed by Karolinska Institutet and receives funding through the Swedish Research Council under the grant no 2017-00641. TwinGene was supported by grants from the Swedish Research Council (M-2005-1112), GenomEUtwin (EU/QLRT-2001-01254; QLG2-CT-2002-01254), NIH DK U01-066134, The Swedish Foundation for Strategic Research (SSF). Heart and Lung foundation no. 20070481. Analysis on renal biomarkers was supported by grants from Stockholm County Council, The Swedish Society of Medicine (SLS-412071), the Serafimer Hospital Foundation, Stiftelsen för Njursjuka and The Swedish Kidney Foundation (F2019-0003).

ULSAM The ULSAM study was supported by Wellcome Trust grants WT098017, WT064890, WT090532, Uppsala University, Uppsala University Hospital, the Swedish Research Council and the Swedish Heart-Lung Foundation. Johan Ärnlöv was supported by The Swedish Research Council, Swedish Heart-Lung Foundation, Dalarna University and Uppsala University.

Vanderbilt The data used for the analyses were obtained from Vanderbilt University Medical Center’s BioVU, which is supported by numerous sources: institutional funding, private agencies, and federal grants. These include the

NIH funded Shared Instrumentation Grant S10RR025141; and CTSA grants UL1TR002243, UL1TR000445, and UL1RR024975. Genomic data are also supported by investigator-led projects that include U01HG004798, R01NS032830, RC2GM092618, P50GM115305, U01HG006378, U19HL065962, R01HD074711; and additional funding sources listed at https://victr.vanderbilt.edu/pub/biovu/. Jacklyn N. Hellwege is supported by the Vanderbilt Molecular and Genetic Epidemiology of Cancer training program, funded by T32CA160056.

VIKING The Viking Health Study – Shetland (VIKING) was supported by the MRC Human Genetics Unit quinquennial programme grant “QTL in Health and Disease”. DNA extractions and genotyping were performed at the Edinburgh Clinical Research Facility, University of Edinburgh. We acknowledge the contributions of the research nurses in Shetland, the administrative team in Edinburgh, the people of Shetland, and the data analysts, in particular Dr Thibaud Boutin for the genotype imputation to the HRC reference panel.

WGHS The Women's Genome Health Study (WGHS) is supported by the National Heart, Lung, and Blood Institute (HL043851 and HL080467) and the National Cancer Institute (CA047988 and UM1CA182913) with funding for genotyping provided by Amgen.

YFS The Young Finns Study has been financially supported by the Academy of Finland: grants 322098, 286284, 134309 (Eye), 126925, 121584, 124282, 129378 (Salve), 117787 (Gendi), and 41071 (Skidi); the Social Insurance Institution of Finland; Competitive State Research Financing of the Expert Responsibility area of Kuopio, Tampere and Turku University Hospitals (grant X51001); Juho Vainio Foundation; Paavo Nurmi Foundation; Finnish Foundation for Cardiovascular Research ; Finnish Cultural Foundation; The Sigrid Juselius Foundation; Tampere Tuberculosis Foundation; Emil Aaltonen Foundation; Yrjö Jahnsson Foundation; Signe and Ane Gyllenberg Foundation; Diabetes Research Foundation of Finnish Diabetes Association; EU Horizon 2020 (grant 755320 for TAXINOMISIS and grant 848146 for To Aition); European Research Council (grant 742927 for MULTIEPIGEN project); Tampere University Hospital Supporting Foundation and Finnish Society of Clinical Chemistry. We thank the teams that collected data at all measurement time points; the persons who participated as both children and adults in these longitudinal studies; and biostatisticians Irina Lisinen, Johanna Ikonen, Noora Kartiosuo, Ville Aalto, and Jarno Kankaanranta for data management and statistical advice.

Supplementary Note 5. VA Million Veteran Program: Core Acknowledgement for Publications (Updated April 6, 2020).

MVP Executive Committee

- Co-Chair: J. Michael Gaziano, M.D., M.P.H.
- Co-Chair: Sumitra Muralidhar, Ph.D.
- Rachel Ramoni, D.M.D., Sc.D., Chief VA Research and Development Officer
- Jean Beckham, Ph.D.
- Kyong-Mi Chang, M.D.
- Christopher J. O'Donnell, M.D., M.P.H.
- Philip S. Tsao, Ph.D.
- James Breeling, M.D., Ex-Officio
- Grant Huang, Ph.D., Ex-Officio
- JP Casas Romero, M.D., Ph.D., Ex-Officio

MVP Program Office

- Sumitra Muralidhar, Ph.D.
- Jennifer Moser, Ph.D.

MVP Recruitment/Enrollment

- Recruitment/Enrollment Director/Deputy Director, Boston – Stacey B. Whitbourne, Ph.D.; Jessica V. Brewer, M.P.H.
- MVP Coordinating Centers
    - Clinical Epidemiology Research Center (CERC), West Haven – Mihaela Aslan, Ph.D.
    - Cooperative Studies Program Clinical Research Pharmacy Coordinating Center, Albuquerque – Todd Connor, Pharm.D.; Dean P. Argyres, B.S., M.S.
    - Genomics Coordinating Center, Palo Alto – Philip S. Tsao, Ph.D.
    - MVP Boston Coordinating Center, Boston - J. Michael Gaziano, M.D., M.P.H.
    - MVP Information Center, Canandaigua – Brady Stephens, M.S.
- VA Central Biorepository, Boston – Mary T. Brophy M.D., M.P.H.; Donald E. Humphries, Ph.D. Luis E. Selva, Ph.D.
- MVP Informatics, Boston – Nhan Do, M.D.; Shahpoor Shayan
- MVP Data Operations/Analytics, Boston – Kelly Cho, Ph.D.

MVP Science

- Science Operations – Christopher J. O'Donnell, M.D., M.P.H
- Genomics Core - Christopher J. O'Donnell, M.D., M.P.H.; Saiju Pyarajan Ph.D.; Philip S. Tsao, Ph.D.
- Phenomics Core- Kelly Cho, M.P.H, Ph.D.
- Data and Computational Sciences – Saiju Pyarajan, Ph.D.
- Statistical Genetics – Elizabeth Hauser, Ph.D.; Yan Sun, Ph.D.; Hongyu Zhao, Ph.D.

Current MVP Local Site Investigators

- Atlanta VA Medical Center (Peter Wilson, M.D.)
- Bay Pines VA Healthcare System (Rachel McArdle, Ph.D.)

- Birmingham VA Medical Center (Louis Dellitalia, M.D.)
- Central Western Massachusetts Healthcare System (Kristin Mattocks, Ph.D., M.P.H.)
- Cincinnati VA Medical Center (John Harley, M.D., Ph.D.)
- Clement J. Zablocki VA Medical Center (Jeffrey Whittle, M.D., M.P.H.)
- VA Northeast Ohio Healthcare System (Frank Jacono, M.D.)
- Durham VA Medical Center (Jean Beckham, Ph.D.)
- Edith Nourse Rogers Memorial Veterans Hospital (John Wells., Ph.D.)
- Edward Hines, Jr. VA Medical Center (Salvador Gutierrez, M.D.)
- Veterans Health Care System of the Ozarks (Gretchen Gibson, D.D.S., M.P.H.)
- Fargo VA Health Care System (Kimberly Hammer, Ph.D.)
- VA Health Care Upstate New York (Laurence Kaminsky, Ph.D.)
- New Mexico VA Health Care System (Gerardo Villareal, M.D.)
- VA Boston Healthcare System (Scott Kinlay, M.B.B.S., Ph.D.)
- VA Western New York Healthcare System (Junzhe Xu, M.D.)
- Ralph H. Johnson VA Medical Center (Mark Hamner, M.D.)
- Columbia VA Health Care System (Roy Mathew, M.D.)
- VA North Texas Health Care System (Sujata Bhushan, M.D.)
- Hampton VA Medical Center (Pran Iruvanti, D.O., Ph.D.)
- Richmond VA Medical Center (Michael Godschalk, M.D.)
- Iowa City VA Health Care System (Zuhair Ballas, M.D.)
- Eastern Oklahoma VA Health Care System (Douglas Ivins, M.D.)
- James A. Haley Veterans' Hospital (Stephen Mastorides, M.D.)
- James H. Quillen VA Medical Center (Jonathan Moorman, M.D., Ph.D.)
- John D. Dingell VA Medical Center (Saib Gappy, M.D.)
- Louisville VA Medical Center (Jon Klein, M.D., Ph.D.)
- Manchester VA Medical Center (Nora Ratcliffe, M.D.)
- Miami VA Health Care System (Hermes Florez, M.D., Ph.D.)
- Michael E. DeBakey VA Medical Center (Olaoluwa Okusaga, M.D.)
- Minneapolis VA Health Care System (Maureen Murdoch, M.D., M.P.H.)
- N. FL/S. GA Veterans Health System (Peruvemba Sriram, M.D.)
- Northport VA Medical Center (Shing Shing Yeh, Ph.D., M.D.)
- Overton Brooks VA Medical Center (Neeraj Tandon, M.D.)
- Philadelphia VA Medical Center (Darshana Jhala, M.D.)
- Phoenix VA Health Care System (Samuel Aguayo, M.D.)
- Portland VA Medical Center (David Cohen, M.D.)
- Providence VA Medical Center (Satish Sharma, M.D.)
- Richard Roudebush VA Medical Center (Suthat Liangpunsakul, M.D., M.P.H.)
- Salem VA Medical Center (Kris Ann Oursler, M.D.)
- San Francisco VA Health Care System (Mary Whooley, M.D.)
- South Texas Veterans Health Care System (Sunil Ahuja, M.D.)
- Southeast Louisiana Veterans Health Care System (Joseph Constans, Ph.D.)
- Southern Arizona VA Health Care System (Paul Meyer, M.D., Ph.D.)
- Sioux Falls VA Health Care System (Jennifer Greco, M.D.)
- St. Louis VA Health Care System (Michael Rauchman, M.D.)
- Syracuse VA Medical Center (Richard Servatius, Ph.D.)
- VA Eastern Kansas Health Care System (Melinda Gaddy, Ph.D.)
- VA Greater Los Angeles Health Care System (Agnes Wallbom, M.D., M.S.)
- VA Long Beach Healthcare System (Timothy Morgan, M.D.)

- VA Maine Healthcare System (Todd Stapley, D.O.)
- VA New York Harbor Healthcare System (Scott Sherman, M.D., M.P.H.)
- VA Pacific Islands Health Care System (George Ross, M.D.)
- VA Palo Alto Health Care System (Philip Tsao, Ph.D.)
- VA Pittsburgh Health Care System (Patrick Strollo, Jr., M.D.)
- VA Puget Sound Health Care System (Edward Boyko, M.D.)
- VA Salt Lake City Health Care System (Laurence Meyer, M.D., Ph.D.)
- VA San Diego Healthcare System (Samir Gupta, M.D., M.S.C.S.)
- VA Sierra Nevada Health Care System (Mostaqul Huq, Pharm.D., Ph.D.)
- VA Southern Nevada Healthcare System (Joseph Fayad, M.D.)
- VA Tennessee Valley Healthcare System (Adriana Hung, M.D., M.P.H.)
- Washington DC VA Medical Center (Jack Lichy, M.D., Ph.D.)
- W.G. (Bill) Hefner VA Medical Center (Robin Hurley, M.D.)
- White River Junction VA Medical Center (Brooks Robey, M.D.)
- William S. Middleton Memorial Veterans Hospital (Robert Striker, M.D., Ph.D.)

Supplementary Note 6. LifeLines group author genetics.

Raul Aguirre-Gamboa (1), Patrick Deelen (1), Lude Franke (1), Jan A Kuivenhoven (2), Esteban A Lopera Maya (1), Ilja M Nolte (3), Serena Sanna (1), Harold Snieder (3), Morris A Swertz (1), Judith M Vonk (3), Cisca Wijmenga (1)

(1) Department of Genetics, University of Groningen, University Medical Center Groningen, The Netherlands

(2) Department of Pediatrics, University of Groningen, University Medical Center Groningen, The Netherlands

Department of Epidemiology, University of Groningen, University Medical Center Groningen, The Netherlands

Supplementary References

1. Groopman, E. E. *et al.* Diagnostic Utility of exome sequencing for kidney disease. *N. Engl. J. Med.* (2019). doi:10.1056/NEJMoa1806891
2. Stanzick, K. J. *et al.* Discovery and prioritization of variants and genes for kidney function in >1.2 million individuals. *Nat. Commun. 2021 121* **12**, 1–17 (2021).
3. Wuttke, M. *et al.* A catalog of genetic loci associated with kidney function from analyses of a million individuals. *Nat. Genet.* **51**, 957–972 (2019).
4. Rentzsch, P., Witten, D., Cooper, G. M., Shendure, J. & Kircher, M. CADD: Predicting the deleteriousness of variants throughout the human genome. *Nucleic Acids Res.* (2019). doi:10.1093/nar/gky1016
5. Gillies, C. E. *et al.* An eQTL Landscape of Kidney Tissue in Human Nephrotic Syndrome. *Am. J. Hum. Genet.* (2018). doi:10.1016/j.ajhg.2018.07.004
6. Aguet, F. *et al.* Genetic effects on gene expression across human tissues. *Nature* **550**, 204–213 (2017).
7. Hamosh, A., Scott, A. F., Amberger, J., Valle, D. & McKusick, V. A. Online Mendelian Inheritance in Man (OMIM). *Hum. Mutat.* (2000). doi:10.1002/(SICI)1098-1004(200001)15:1<57::AID-HUMU12>3.0.CO;2-G
8. C, F. *et al.* Log-transformation and its implications for data analysis. *Shanghai Arch. psychiatry* **26**, 105–109 (2014).
9. Keller, M. C. Gene × environment interaction studies have not properly controlled for potential confounders: The problem and the (simple) solution. *Biol. Psychiatry* **75**, 18–24 (2014).
10. Winkler, T. W. *et al.* Approaches to detect genetic effects that differ between two strata in genome-wide meta-analyses: Recommendations based on a systematic evaluation. *PLoS One* **12**, (2017).
