# Supplementary material for: Differential and shared genetic effects on kidney function between diabetic and non-diabetic individuals
Source: Commun Biol. 2022 Jun 13;5:580. doi: 10.1038/s42003-022-03448-z (PMC9192715; doi:10.1038/s42003-022-03448-z)
Supplement: Supplementary file 3 — Description of Additional Supplementary Files [file 42003_2022_3448_MOESM3_ESM.pdf]

Description of Additional Supplementary Files

File name: Supplementary Data 1
Description: Study descriptives.

File name: Supplementary Data 2
Description: Lead variants of seven difference loci identified by difference test approach or overall+difference approach.

File name: Supplementary Data 3
Description: 610 variants with significant eGFR association overall.

File name: Supplementary Data 4
Description: Sensitivity analyses based on GxC interaction models in UKB for 7 difference locus lead variants.

File name: Supplementary Data 5
Description: 32 novel loci identified by joint test or stratified test approaches.

File name: Supplementary Data 6
Description: CUBN region lookup for UACR association.

File name: Supplementary Data 7
Description: Comparison of GxDM and GxHbA1c interaction.

File name: Supplementary Data 8
Description: Diabetes and Glycemic traits GWAS Lookup for the 11 difference locus lead variants.

File name: Supplementary Data 9
Description: Opentargets PheWAS for the lead variants from 11 difference loci.

File name: Supplementary Data 10
Description: Lead variants of 29 genome-wide signficant eGFR loci identified in DM-only GWAS.

File name: Supplementary Data 11
Description: Genepriotisation (GPS) for the 32 novel eGFR loci.

File name: Supplementary Data 12
Description: 99% credible set variants for the 34 signals at 32 novel eGFR loci.

File name: Supplementary Data 13
Description: Annotation of 99% credible set variants with CADD>= 15 residing within gene at 32 novel eGFR loci.

File name: Supplementary Data 14

Description: Significant eQTLs in NEPTUNE glomerular or tubulo-interstitial tissue for the 99% credible set variants at novel eGFR loci.

File name: Supplementary Data 15
Description: Significant eQTLs in GTEx kidney tissue for the 99% credible set variants at novel eGFR loci.
