# Supplementary material for: Differential and shared genetic effects on kidney function between diabetic and non-diabetic individuals
Source: Commun Biol. 2022 Jun 13;5:580. doi: 10.1038/s42003-022-03448-z (PMC9192715; doi:10.1038/s42003-022-03448-z)
Supplement: Supplementary file 19 — Reporting summary [file 42003_2022_3448_MOESM19_ESM.pdf]

nature portfolio

Corresponding author(s): Thomas Winkler

Last updated by author(s): Apr 1, 2022

Reporting Summary

Nature Portfolio wishes to improve the reproducibility of the work that we publish. This form provides structure for consistency and transparency in reporting. For further information on Nature Portfolio policies, see our Editorial Policies and the Editorial Policy Checklist.

Statistics

For all statistical analyses, confirm that the following items are present in the figure legend, table legend, main text, or Methods section.

n/a	Confirmed	
☐	☒	The exact sample size (*n*) for each experimental group/condition, given as a discrete number and unit of measurement
☐	☒	A statement on whether measurements were taken from distinct samples or whether the same sample was measured repeatedly
☐	☒	The statistical test(s) used AND whether they are one- or two-sided<br>*Only common tests should be described solely by name; describe more complex techniques in the Methods section.*
☐	☒	A description of all covariates tested
☐	☒	A description of any assumptions or corrections, such as tests of normality and adjustment for multiple comparisons
☐	☒	A full description of the statistical parameters including central tendency (e.g. means) or other basic estimates (e.g. regression coefficient) AND variation (e.g. standard deviation) or associated estimates of uncertainty (e.g. confidence intervals)
☐	☒	For null hypothesis testing, the test statistic (e.g. *F*, *t*, *r*) with confidence intervals, effect sizes, degrees of freedom and *P* value noted<br>*Give P values as exact values whenever suitable.*
☒	☐	For Bayesian analysis, information on the choice of priors and Markov chain Monte Carlo settings
☒	☐	For hierarchical and complex designs, identification of the appropriate level for tests and full reporting of outcomes
☐	☒	Estimates of effect sizes (e.g. Cohen's *d*, Pearson's *r*), indicating how they were calculated

Our web collection on statistics for biologists contains articles on many of the points above.

Software and code

Policy information about availability of computer code

Data collection	Various standard GWAS tools were used by the different studies to conduct diabetes-status specific eGFR GWAS. Data was collected centrally on a ftp site and QC'ed using GWAtoolbox (https://academic.oup.com/bioinformatics/article/28/3/444/189687) and EasyQC (https://www.nature.com/articles/nprot.2014.071).
Data analysis	Study-specific GWAS were meta-analysed by diabetes-status using metal (https://www.ncbi.nlm.nih.gov/pmc/articles/PMC2922887/) and evaluated using EasyStrata (https://academic.oup.com/bioinformatics/article/31/2/259/2365778).

For manuscripts utilizing custom algorithms or software that are central to the research but not yet described in published literature, software must be made available to editors and reviewers. We strongly encourage code deposition in a community repository (e.g. GitHub). See the Nature Portfolio guidelines for submitting code & software for further information.

Data

Policy information about availability of data

All manuscripts must include a data availability statement. This statement should provide the following information, where applicable:

- Accession codes, unique identifiers, or web links for publicly available datasets
- A description of any restrictions on data availability
- For clinical datasets or third party data, please ensure that the statement adheres to our policy

Summary genetic association results for the DM-status specific meta-analyses for log(eGFRcrea) can be downloaded from https://ckdgen.imbi.uni-freiburg.de/. All other data is available from the corresponding author on reasonable request.

Field-specific reporting

Please select the one below that is the best fit for your research. If you are not sure, read the appropriate sections before making your selection.

☒ Life sciences ☐ Behavioural & social sciences ☐ Ecological, evolutionary & environmental sciences

For a reference copy of the document with all sections, see nature.com/documents/nr-reporting-summary-flat.pdf

Life sciences study design

All studies must disclose on these points even when the disclosure is negative.

Sample size	We collected as much GWAS for eGFR as possible within the Chronic Kidney Disease Genetics Consortium yielding a total GWAS sample size of 178,691 individuals with diabetes and 1,296,113 individuals without diabetes.
Data exclusions	We excluded variants with low imputation quality, Info<0.6, or rare variants with minor allele frequency, MAF<0.1%.
Replication	Our data was collected in two independent stages. We used stage 1 as dicovery and perfomerd formal replication using a Bonferroni-correction in stage 2. In addition, we used the combined stage 1+2 for identification to maximize power for the identification.
Randomization	Randomization was not relevant. Our main analyses were GWAS based on linear regression correcting for age, sex and principal components.
Blinding	Blinding was not relevant. Our main analyses were GWAS based on linear regression correcting for age, sex and principal components.

Reporting for specific materials, systems and methods

We require information from authors about some types of materials, experimental systems and methods used in many studies. Here, indicate whether each material, system or method listed is relevant to your study. If you are not sure if a list item applies to your research, read the appropriate section before selecting a response.

Materials & experimental systems

n/a	Involved in the study	
☒	☐	Antibodies
☒	☐	Eukaryotic cell lines
☒	☐	Palaeontology and archaeology
☒	☐	Animals and other organisms
☐	☒	Human research participants
☒	☐	Clinical data
☒	☐	Dual use research of concern

Methods

n/a	Involved in the study	
☒	☐	ChIP-seq
☒	☐	Flow cytometry
☒	☐	MRI-based neuroimaging

Human research participants

Policy information about studies involving human research participants

Population characteristics	We include standard GWAS based on linear regression from >72 individual studies. Detailled study descriptives are shown in Supplementary Data 1.
Recruitment	We include standard GWAS based on linear regression from >72 individual studies. Detailled study descriptives are shown in Supplementary Data 1.
Ethics oversight	Each study has its own ethical board that confirmed our study.

Note that full information on the approval of the study protocol must also be provided in the manuscript.
